# PRIMA: a rapid and cost-effective genotyping method to detect single-nucleotide differences using probe-induced heteroduplexes

**DOI:** 10.1038/s41598-021-99641-x

**Published:** 2021-10-24

**Authors:** Hiroyuki Kakui, Misako Yamazaki, Kentaro K. Shimizu

**Affiliations:** 1 Department of Evolutionary Biology and Environmental Studies, University of Zurich, 8057 Zurich, Switzerland; 2 Kihara Institute of Biological Research, Yokohama City University, Yokohama, 244-0813 Japan

**Keywords:** Genotyping and haplotyping, Genetic engineering, PCR-based techniques, Genotype

## Abstract

Targeted mutagenesis by programmable site-specific nucleases like CRISPR typically produce 1-base pair (bp) insertion or deletion (indel) mutations. Although several methods have been developed to detect such 1-bp indels, each method has pros and cons in terms of cost and/or resolution. Heteroduplex mobility assay (HMA) is a traditional technique detecting small base pair differences but it has a limited resolution of mutation size and the band patterns are often complex. Here, we developed a new method called PRIMA (Probe-Induced HMA) using a short single-stranded DNA molecule as a probe in HMA. By utilizing a 40-mer probe containing a 5-nucleotide deletion, we assessed the mobility of a heteroduplex with a target DNA fragment from a plant, bacterium, and human. This method allowed us to detect a 1-bp indel mutation consistently. We also showed that SNPs can be detected using PRIMA. PRIMA provides a rapid and cost-effective solution for the genotyping.

## Introduction

There is an increasing demand in molecular biology to detect 1-bp differences because of the recent advancement of gene-editing technology (ZFN/TALEN/CRISPR) based on double-strand breaks (DSBs). These DSBs can stimulate non-homologous end joining (NHEJ) at the targeted genome sequence and often produce a 1-bp insertion or deletion (indel) mutation^1–5^. Of interest to researchers are 1-bp indel mutations to obtain frameshift null mutants to study phenotypic effects for biological, medical, and agricultural interests. To identify such mutations from a screening population, a large number of samples have to be analyzed. Once a mutation is identified, further genotyping of homozygotes and heterozygotes may be required for subsequent analysis. Such experiments have been performed in many organisms, and the requirement for experiments would be increased when many genes or many homeologs in polyploid species are manipulated^6–14^. Methods detecting small base pair differences have been developed by many researchers^15^, for example, Sanger or deep sequencing, restriction fragment length polymorphism (RFLP) analysis^16^, heteroduplex mobility assay (HMA)^17–20^, DNA melting analysis^21^, T7 endonuclease I assay^22^, Cel-1 assay^23^, fluorescent polymerase chain reaction (PCR)^24^, and RNA-guided engineered nuclease (RGEN)-RFLP^25^. Each technique has advantages and disadvantages in terms of budget and time requirements. For example, Sanger or deep sequencing can identify DNA sequences at a 1-bp resolution but is relatively expensive and slow. RFLP analysis can achieve 1-bp resolution but is not suitable for the screening of new mutations because the mutation sites need to be known to design the experiment. DNA melting analysis, T7 endonuclease I assay, Cel-1 assay, fluorescent PCR, and RGEN-RFLP are not always successful in obtaining 1-bp resolution or need special chemicals/proteins/devices, raising the cost of experiments.

**Figure 1:**
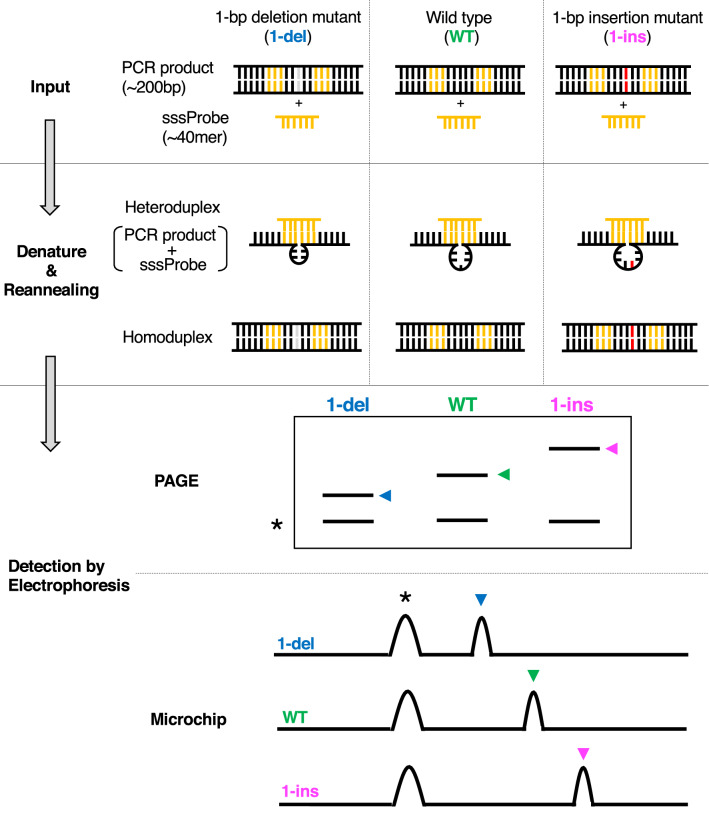
Overview of PRIMA (Probe-Induced Heteroduplex Mobility Assay). Input step: PCR products are mixed with a short single-strand probe (sssDNA). Mutant sequences have a small indel (1-bp deletion sequence on the left side, or insertion sequence on the right side). Yellow lines indicate the binding region of the PCR product and the probe. Gray and red lines indicate deleted and inserted nucleotides, respectively. Denature and Reannealing step: Heteroduplexes are produced after denaturing and reannealing. In this figure, 4-nt, 5-nt, or 6-nt bulge structures are produced from 1-bp deletion (1-del), wild type (WT), and 1-bp insertion (1-ins) sequences, respectively. Detection step: heteroduplexes are detected using polyacrylamide gel electrophoresis (PAGE) or a microchip electrophoresis system. Asterisks indicate homoduplex signals. Blue, green, and magenta arrowheads indicate heteroduplex signals from 1-bp deletion, Wild type, and 1-bp insertion, respectively. Note that the schematic figures of PAGE and microchip represent simplified pictures, although more than a single extra signal possibly due to more complex DNA structure may be observed (see text).

HMA is a standard method for many laboratories and has been used to detect small base pair differences, typically down to three or more base pair differences^15,17–20^. Electron microscopic study showed that a heteroduplex DNA between wild-type and mutant sequences with a small base pair difference produced a bulge structure due to the looped-out bases^26^. When examined by electrophoreses, two extra bands corresponding to each DNA strand are typically observed as expected, although only one or more than three extra bands are often observed^27^. Such complex patterns were attributed to different conformations or different annealing possibilities of the single-stranded DNA, and may depend on experimental settings^27^. Importantly, except for a few cases^28^, it is normally impossible to distinguish 1-bp differences using HMA unless producing a double-stranded DNA probe with mutations (called improved heteroduplex analysis (iHDA)^29^). Here, we developed a generally applicable method to detect a 1-bp difference using a short single-strand DNA (sssDNA) probe and called it PRIMA (Probe-Induced HMA, Fig. 1 and Supplementary Fig. S1, right). To explain PRIMA, we first show HMA results (Supplementary Fig. S1, left), and then we explain prePRIMA, which is the precursive method of PRIMA (Supplementary Fig. S1, middle). Finally, we describe PRIMA, which has 1-bp resolution and is a fast method capable of genotyping.

## Results and discussion

### Traditional HMA is not adequate to detect 1-bp indels

HMA consists of four simple steps: (1) PCR (target DNA fragment amplification), (2) denaturation, (3) reannealing, and (4) electrophoresis (Supplementary Fig. S1, left). When two types of sequences exist in step 3 (e.g., for a mutant with a 1-bp deletion and WT in Supplementary Fig. S1, left), reannealed sequences create so-called heteroduplex DNA. Heteroduplex DNA between wild-type and mutant sequences induces a bulge structure due to the looped-out bases (bulges are shown by the arrow in Supplementary Fig. S1; left “Reannealing”). If heteroduplex DNA has enough bulge length (typically a length difference of three or more base pair indels), the mobility of heteroduplex DNA tends to be slower than that of the homoduplex DNA. These heteroduplex mobility shifts may be detected using polyacrylamide gel electrophoresis (PAGE) or a microchip electrophoresis system^17–20^. However, heteroduplex mobility shifts are usually not observed for a 1-bp indel (Supplementary Fig. S1, left, "Electrophoresis").

We tested a series of deletion sequences to characterize the resolution of HMA in a microchip electrophoresis system (MCE-202 MultiNA, Shimadzu, Japan, hereafter called MultiNA) using a DNA-500 reagent kit. MultiNA detects nucleic acid fragments based on their mobility and is also applicable to detect heteroduplex mobility shifts. The pattern of DNA mobility is displayed as an electropherogram (depicted schematically in Supplementary Fig. S1 bottom, and shown in, e.g., Supplementary Fig. S2). The X- and Y-axes correspond to the DNA size estimated from the mobility, and to the signal intensity that reflects the DNA quantity, respectively. Using PCR, we prepared wild-type and mutant sequences carrying different lengths of deletions, i.e., wild-type to 7-bp deleted sequences separately. We call them WT (from wild type), 1-del (from 1-bp deleted sequence) to 7-del (7-bp deleted sequence), respectively. Every target fragment was subsequently mixed with WT. These mixtures were denatured and reannealed to induce the heteroduplex complexes. Finally, the mobility of the reannealed DNA mixtures was analyzed using the MultiNA (Supplementary Fig. S2). Two different heteroduplex peaks were often observed by HMA, which may be consistent with the presence of two heteroduplex structures derived from each DNA strand (Supplementary Fig. S1, S2, left “Reannealing, Heteroduplex”). Similar to the results shown previously^20^, we could not detect the 1-bp bulge as a heteroduplex peak (“1-del + WT” in Supplementary Fig. S2). Heteroduplex peaks with a 2-bp or 3-bp bulge were often not clearly observed, also consistent with previous studies (“2-del + WT” in Supplementary Fig. S2a and “3-del + WT” in 2b)^18,19^. By contrast, 4-bp or larger bulges resulted in the patterns of peaks that are distinguishable from those of WT homoduplex and 1-bp or 2-bp bulged peaks. Importantly, mixtures of WT and 4-del, 5-del, or 6-del showed the heteroduplex peaks clearly distinguished from each other (“4-del + WT”, “5-del + WT”, and “6-del + WT” in Supplementary Fig. S2). This finding inspired the idea of prePRIMA (next paragraph).

We examined further the effect of target fragment sizes (Supplementary Fig. S3). Target fragments with about 200-bp size worked well to detect different heteroduplex peaks among 4-bp to 7-bp bulge fragments (Supplementary Fig. S2). Shorter target fragments showed less obvious mobility shift of heteroduplex peaks compared with 200-bp DNA fragments and it was difficult to distinguish heteroduplex peaks of 4-bp to 6-bp bulge (Supplementary Fig. S3b). In contrast, longer target fragments produced a larger heteroduplex mobility shift, which often could not be analyzed confidently because of the overlap with the upper marker (UM) (“5-del + WT”, “6-del + WT”, and “7-del + WT” in Supplementary Fig. S3c). Taken together, we found that around 200-bp is a suitable length with DNA-500 reagent kit in MultiNA for the fine resolution of HMA produced by 4-bp to 6-bp bulges.

### Mixing a 5-bp deletion probe enabled the 1-bp resolution

The HMA results described above inspired a strategy to detect the 1-bp difference between WT and the 1-bp deletion sequence, or that between WT and 1-bp insertion sequence. We were able to distinguish the heteroduplex patterns of HMA in the case of 4-bp versus 5-bp bulge, and 5-bp versus 6-bp bulge (Supplementary Fig. S2), in contrast to the case of no- versus 1-bp bulge. Therefore, if we use a sequence with a 5-bp deletion as a probe, we should be able to distinguish 1-bp deletion (4-bp bulge with the probe), WT sequence (5-bp bulge with the probe), and 1-bp insertion (6-bp bulge with the probe). We tested this idea using five genes, which were either from *Arabidopsis thaliana,* bacteria, or humans. We used a target fragment size from 200- to 280-bp because of our HMA results described above (Supplementary Table S1). Indeed, we clearly identified the 1-bp insertion or deletion in all cases we tested (Supplementary Fig. S4). We called this technique prePRIMA (precursive method of PRIMA; Supplementary Fig. S1 middle, prePRIMA).

Next, we considered the bulge position for designing probes. In HMA, it is known that the mobility shift is clearer when the bulge position is closer to the center of its target fragment^20^. In prePRIMA, similarly, we found that the mobility shifts were clear when the probes were designed to set the bulge region in the middle of the target fragment (Supplementary Fig. S5).

### PRIMA: short single-strand DNA was adequate as a probe

Although prePRIMA and iHDA are 1-bp resolution methods, a major drawback is an effort or cost to produce a probe with a few-bp deletion or insertion in the middle of a 200-bp target fragment. Unless such variants are already available, time and/or expense is required to make the probe by a two-step PCR or by cloning^29,30^. Alternatively, it is possible to purchase synthesized DNA, although the fee for a 200-nucleotide (nt) oligo is relatively expensive. To overcome this drawback, we tested the possibility of using short single-stranded DNAs as probes. We designed relatively short single-strand DNAs (sssDNA), which might be sufficient to produce heteroduplexes with looped-out bases, thus enabling detection of a 1-bp indel (Supplementary Fig. S6). In addition to 60-mer and 50-mer strands (Supplementary Fig. S6b,c), we found that sssDNA of 40-mer was sufficient to discriminate the 1-bp different sequences from their WT (Supplementary Fig. S6d). Longer probes tended to show stronger heteroduplex signals (Supplementary Fig. S6). Taken together, we recommend 40-mer DNA for the probe. From these findings, we called our method PRIMA (Probe-Induced Heteroduplex Mobility Assay with sssDNA; Fig. 1 and Supplementary Fig. S1 right, PRIMA).

### PRIMA distinguishes 1-bp difference in various sequences

**Figure 2:**
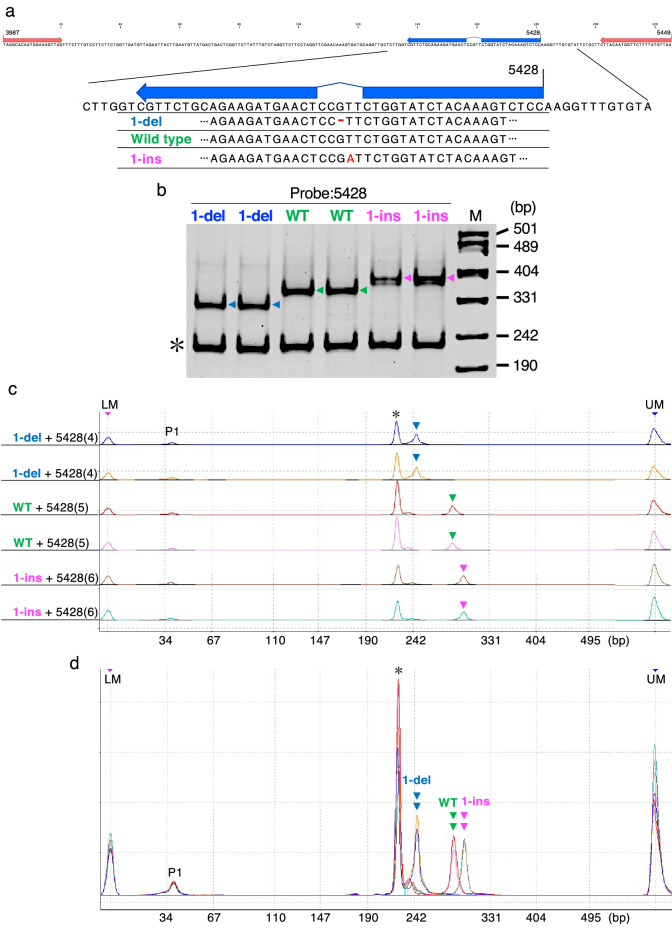
Detection of heteroduplex signals with PRIMA. Different heteroduplex signals were detected from wild-type and 1-bp indel sequences from *RDP1* gene by PRIMA. Partial *RDP1* sequence, mutation sequences, primers, and probe are shown in (**a**) and Supplementary Table S1. Amplified DNA fragments were subjected to PRIMA analysis and electrophoresed using a PAGE (**b**) and microchip system (**c**,**d**). *WT* wild-type sequence, *1-del* 1-bp deletion sequence, *1-ins* 1-bp insertion sequence, *asterisk* homoduplex signal. Blue, green, and magenta arrowhead indicate heteroduplex signals from the 1-del, WT, and 1-ins, respectively. Microchip results were shown by individual views (**c**) and overlay view (**d**). Abbreviations in microchip results (**c**,**d**) are as follows: *LM* lower marker, *UM* upper marker, P1 considered to be leftover of the unbound probe. Bulge lengths between target DNA and probe are shown in brackets next to sample names (**c**). Dashed lines in the background of the chromatograms were automatically added by the software (MultiNA viewer). Abbreviation M in PAGE indicates a pUC19/*Msp*I marker.

To evaluate the PRIMA method, we first analyzed wild-type and cloned 1-bp deletion/insertion sequences of the *RDP1* gene of the plant *Arabidopsis thaliana* (Fig. 2a). Distinguishable heteroduplex signals were obtained from PAGE (Fig. 2b) and microchip system (Fig. 2c). Overlay view of the microchip system clearly showed different peak positions between the different heteroduplex structure derived from 1-del, WT, and 1-ins, in contrast to the same peak positions of replicated samples (Fig. 2d). We further performed PRIMA with wild type and 1-bp indel mutants from human, bacteria, and *A. thaliana*. We tested both strand probes (the same region but bind reverse complementary strands; Supplementary Table S1). We confirmed that heteroduplex signals were different between WT and 1-indel mutant sequences in all samples in both the microchip system and PAGE (Supplementary Fig. S7). As explained above, the band patterns can be simple or complex^27^. In a typical case using microchip system, a single heteroduplex signal was observed in a different position between WT and mutant as expected for the usage of a single-strand DNA probe. In addition, two or more extra signals were often observed, similar to observed multi-HMA signals attributed to different conformations or different annealing possibilities^27^ (Supplementary Fig. S7a,c,f,g,i,m,o) or strong non-specific signals (Supplementary Fig. S7i,l,o). PAGE tended to show more non-specific or indistinguishable signals than the microchip system (Supplementary Fig. S7). These additional bands may represent more complex DNA structures such as three chain combination, which may be assessed by NMR or by a high-resolution microscope^31–34^. We detected different mobility of heteroduplex signals if we compared the results with reverse complementary probes each other (e.g., for a probe 5610 and 5611 in Supplementary Fig. S7a–c). In addition, we found a case in which one of the two probes did not give heteroduplex signals depending on the size of the target fragments (Supplementary Fig. S8). Thus, we currently recommend testing two complementary probes (both directions in the same region, Supplementary Fig. S8), or modifying the fragment size when the difference was not detectable. In short, the band patterns of WT and mutants were different in all the genes and in both PAGE and microchip results, supporting the general applicability of PRIMA.

### Heteroduplex patterns of PRIMA from a 10-bp deletion to a 10-bp insertion sequences

To determine whether PRIMA can be applied to detect indels of longer than 1-bp, we examined further the heteroduplex signal pattern of PRIMA with a 21-variant series of target sequences of *RDP1*, i.e., from a 10-bp deletion to a 10-bp insertion (Supplementary Fig. S9a). The same probe was used for all the 21 target sequences (probe ID: 5428, Supplementary Table S1). Note that the different bulge structure is formed according to the difference between the target fragments and a probe (for example, 6-nt deletion sequence with the 5-nt deletion probe results in 1-bp difference). Different mobilities were observed between the heteroduplex signals derived from the mutants, compared with the signal derived from the WT using the microchip system and by PAGE (Supplementary Fig. S9). In contrast to the presence of heteroduplex signal with WT, as expected, its absence is the characteristic of 6-, 5-, 4-, 3- or 2-del (Supplementary Fig. S9b) in the microchip system or 5-del or 4-del by PAGE (Supplementary Fig. S9d). The absence suggests that the bulge lengths created by the target fragment and probe sequences are not sufficient to produce the mobility shifts because their template and probe DNA duplex has only from 0 to 3-nt gaps. In addition, two mutant samples showed indistinguishable heteroduplex signals with WT (9-del in MultiNA (Supplementary Fig. S9b) and 10-del in PAGE (Supplementary Fig. S9d)). However, these mutants are able to be distinguished by comparing homoduplex signals (Supplementary Fig. S9e,f). These data indicate that PRIMA is useful to distinguish not only 1-bp indels but also larger indels.

As described above, PRIMA can be tested using either a microchip system or by PAGE. We observed that the positions of heteroduplex peaks are not matched and not directly comparable between detection systems. When we used the same target fragments and the same probes, heteroduplex signals in PAGE were typically detected at larger signal sizes than in the microchip system (Fig. 2, Supplementary Figs S7, S8, S9). Although PAGE tends to provide nonspecific signals that are shared among the lanes with WT and indels (Supplementary Fig. S7f,i,l), this did not affect the detection of the heteroduplex peaks. In short, PAGE has the advantage of a cheaper initial investment, while the microchip system has the advantages of clearer results and high-throughput detection with automated sample handling.

### One-step genotyping of the F2 population including heterozygotes using PRIMA

**Figure 3:**
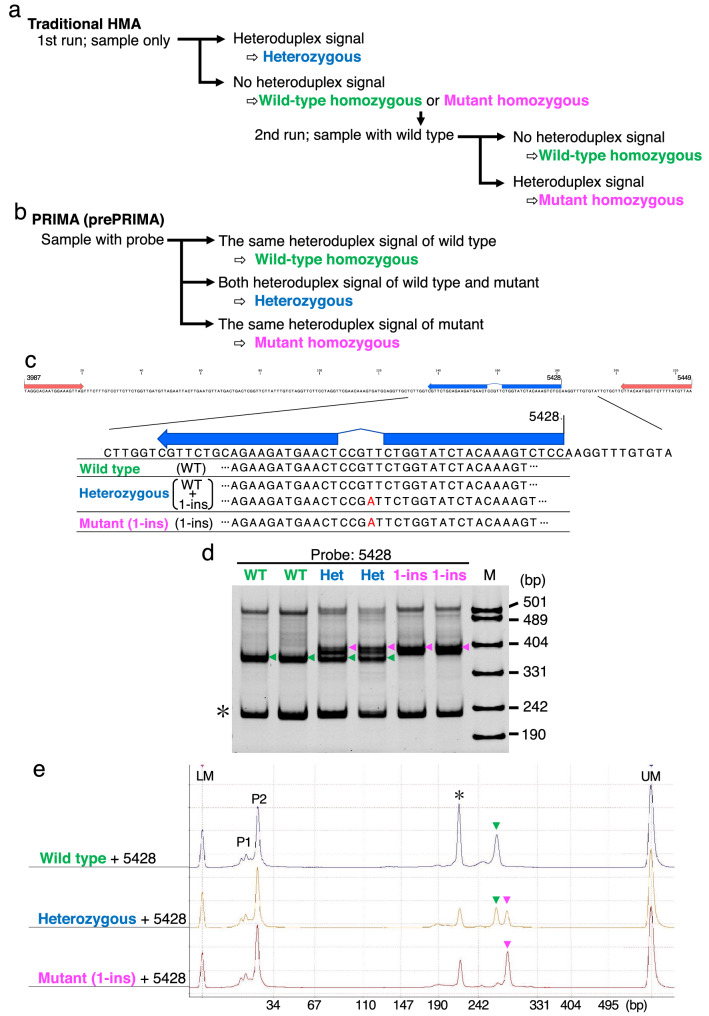
One-step genotyping from the offspring of a heterozygous mutant using PRIMA. Overview of the genotyping steps to distinguish WT homozygous, mutant homozygous, or heterozygous genotypes using HMA (**a**) or PRIMA (**b**). Although HMA needs two runs to distinguish the three genotypes (**a**), PRIMA only needs a single run (**b**). (**c**–**e**) Examples of one-step genotyping using PRIMA. (**c**) Partial *RDP1* sequence and the mutation position are shown. Primers used were the same as in Fig. 2 (3987 × 5449, Supplementary Table S1). The blue arrow indicates the probe sequence. Genotyping results are displayed in (**d**) (PAGE) and (**e**) (Microchip). Asterisks indicate homoduplex peaks. Green and magenta arrowheads indicate heteroduplex peaks derived from the wild-type and mutant sequences, respectively. *WT* wild type, *Het* heterozygous, *1-ins* 1-bp insertion mutant, *M* pUC19/*Msp*I marker, *LM* lower marker, *UM* upper marker, P1 and P2 peak are considered to be leftover primers from the PCR reaction and the unbound probe, respectively.

Once desired 1-bp indel mutations were screened using PRIMA and verified using Sanger sequencing, identification of 1-bp indels is again essential for genotyping in subsequent experiments. For example, wild-type homozygote, heterozygote, and mutant homozygote genotypes are segregating in the progenies derived from a heterozygous diploid parent by selfing. Although traditional HMA has been used for such genotyping^19^, it has two limitations. First, the resolution of HMA is insufficient to detect heteroduplex signals differing in 1-bp as described above (Supplementary Fig. S2). Second, two sets of HMA are necessary to distinguish the three genotypes because another set of HMA by mixing WT target fragments with the samples needs to be run (Fig. 3a) to distinguish two homozygous genotypes. By contrast, PRIMA and prePRIMA distinguish the three genotypes in a single run (Fig. 3b). As described above, using a 5-nt deletion sequence as a probe, heteroduplex peaks derived from WT homozygote or mutant homozygote showed different mobility shifts. Importantly, the heterozygote sample has both peaks and thus can be clearly identified (Fig. 3c–e). Taken together, we concluded PRIMA is suitable for genotyping with a straightforward procedure.

### Heteroduplex patterns of SNPs by PRIMA

In addition to the use of NHEJ caused by DSBs, the recent development of the CRISPR technique enabled precise base-editing resulting in single-nucleotide polymorphisms (SNPs)^35–40^. We tested whether SNPs induce mobility shift using PRIMA. We introduced four-nucleotide variations (A/T/G/C) in *RDP1* (Supplementary Fig. S10a). Using a PRIMA probe, all the four yielded distinguishable heteroduplex peaks by using the microchip system in contrast to unclear results by PAGE (Supplementary Fig. S10b-d). Another PRIMA probe was also successful in yielding different mobility shifts for the four-nucleotide variation using the microchip system (Supplementary Fig. S10e,f), but orders of heteroduplex peaks among the SNPs were different between probes (T < C < A < G by probe 5428 in Supplementary Fig. S10d, C < T < A < G by probe 5531 in Supplementary Fig. S10f). These results suggest that SNPs can be detected by PRIMA, and heteroduplex mobility can be affected not only by bulge length but also by the nucleotide constitution of the bulge and probe position. Further development may broaden its application because SNPs are not only produced by base-editing but also common as natural variations^41,42^.

## Conclusions

In summary, here we established the PRIMA methods to detect a 1-bp difference in genotyping by designing sssDNA for HMA analysis. Targeted mutagenesis protocols by ZFN, TALEN, or CRISPR were established recently^24,43,44^. However, methods for mutation detection or genotyping are not well refined. Among previously developed methods such as RFLP analysis, DNA melting analysis, T7 endonuclease I assay, Cel-1 assay, fluorescent PCR, and RGEN-RFLP, one needs to choose a suitable method on a case-by-case basis depending on budget, time, recognition site of a restriction enzyme in a (putative) mutation site, special devices, or enzymes in the lab. PRIMA, prePRIMA, and iHDA do not need special enzymes or chemicals (Fig. 1 and Supplementary Fig. S1) and provide a fine resolution in the range of 1-bp. In contrast to prePRIMA and iHDA, which require cloning or two-step PCR experiments, PRIMA is a time- and cost-saving protocol with plain results. Furthermore, we described cases of the detection of > 1-bp indels and SNPs using PRIMA. These results suggest that PRIMA has a broad range of applications including the screening of unknown mutations.

## Methods

### Materials

DNA sequences used in this manuscript were from *A. thaliana* (Col-0 accession), bacterial plasmid (pCR4: Thermo Fisher Scientific, MA, USA), and human genomic DNA (Bioline, UK). All information regarding primers, probes, and target sequences is available in Supplementary Table S1.

### Preparation of template sequences

We constructed 10-bp deletion to 10-bp insertion mutation sequences of *RDP1*^45^ using inverse PCR. We prepared 7-bp to 1-bp deletion sequences of *DML1*^46^ by two-step PCR. Primers and templates used are listed in Supplementary Tables S2 and S3, respectively. Ampicillin resistance gene and *alcohol dehydrogenase 1B* were cloned to plasmid and mutation sequences created by cloning with mutated primers (Supplementary Table S4). Target sequences from Ampicillin resistance gene and *alcohol dehydrogenase 1B* partially contain plasmid sequences (Supplementary Table S1). We also used mutated sequences of *RDP1*, AT1G25270, AT2G24440, and AT5G01250, which were created using a CRISPR-Cas9 system^47^. All sequences were confirmed by Sanger sequencing.

### Heteroduplex mobility assay (HMA) and prePRIMA

Target fragments from WT and mutant were amplified using GoTaq polymerase (Promega, USA). We amplified 5-bp deletion probes of prePRIMA using a long primer or 5-bp deletion template (see Supplementary Table S1). Target fragments were mixed with WT fragments (HMA) or 5-bp deletion fragments (prePRIMA) in a 1:1 ratio. Then, to perform denaturation and reannealing reactions we used a temperature cycle as follows: 5 min at 95 °C, cooling to 75 °C, then cooling to 25 °C at 0.2 °C per second. Heteroduplex signals were detected using PAGE or using microchip electrophoresis systems.

### Protocol for the genotyping of CRISPR mutant with PRIMA (Supplementary Fig. S11)

1. Set up a PCR condition based on the target site of genome editing.

Design primers that satisfy the criteria indicated below.

Forward primer position: around 120-bp upstream of the (putative) mutation position.

Reverse primer position: around 120-bp downstream of the (putative) mutation position. The putative mutation position of CRISPR was mainly expected from − 5 to − 1 of the protospacer adjacent motif (PAM) region^35^ (Supplementary Fig. S11).

It is recommended to design these primers for a product size ranging from 200- to 280-bp.

2. Design a probe containing a 5-bp deletion around the (putative) mutation position.

PRIMA works using short single-stranded DNA (sssDNA). We recommend setting 5-bp deletion in the middle of 40-mer sssDNA (20-mer:5-del:20-mer) to maximize hybridization efficiency for both side of sssDNA with the complementary target DNA. We confirmed that at least 40-mer sssDNA probes with 40–60% GC content in the deletion region were adequate to introduce the required conformational change after the reannealing process in step 4 (Supplementary Table S1; Supplementary Fig. S6d). We recommend that the probe starts from − 25 to +20 of the PAM sequence with a 5-bp deletion region (from − 5 to − 1; see Supplementary Fig. S11) because it can cover the major mutation position generated by CRISPR/Cas9^35^.

3. PCR.

Prepare a PCR fragment with a normal PCR protocol using the primers from step 1. This method works without purification step to remove background DNA (e.g., leftover of primers, dNTPs, and template DNA).

4. Preparation of the mixture of PCR product and probe and reannealing.

Mix 9 μL of PCR product from step 3 and 1 μL of 10 μM probe designed in step 2.

Then, perform a denaturation and reannealing reaction using the following conditions: 5 min at 95 °C, cooling to 75 °C, then cooling to 25 °C at 0.2 °C per second.

5. Detection of heteroduplex peak.

Heteroduplex peak(s) can be detected using PAGE or a microchip electrophoresis system.

6. Identification of the exact mutation using Sanger sequencing.

Screened samples can be subjected to Sanger sequencing to identify the exact mutation.

### Electrophoresis using a high-resolution electrophoresis system

MultiNA (MCE-202, Shimadzu, Japan) with a DNA-500 reagent kit or a DNA-1000 reagent kit (Shimadzu) was used. Signals were detected following the manufacturer’s protocol. At least two technical replicates showed the consistent signal patterns for each sample in this study.

### Polyacrylamide gel electrophoresis (PAGE)

An XCell *SureLock* Mini-Cell electrophoresis system (Thermo Fisher Scientific), 6% TBE gel (Thermo Fisher Scientific) and 1 × TBE buffer were used for detecting heteroduplex bands. The gels were electrophoresed for 90 min at 100 V. At least two technical replicates showed the consistent signal patterns for each sample in this study.

## Supplementary Information

Supplementary Information 1.

Supplementary Information 2.

Supplementary Information 3.

Supplementary Information 4.

Supplementary Information 5.
