# Supplementary material for: PRIMA: a rapid and cost-effective genotyping method to detect single-nucleotide differences using probe-induced heteroduplexes
Source: Sci Rep. 2021 Oct 24;11:20741. doi: 10.1038/s41598-021-99641-x (PMC8542037; doi:10.1038/s41598-021-99641-x)
Supplement: Supplementary file 1 — Supplementary Information 1. [file 41598_2021_99641_MOESM1_ESM.pdf]

PRIMA: a rapid and cost-effective genotyping method to detect single-nucleotide differences using probe-induced heteroduplexes

Hiroyuki Kakui, Misako Yamazaki, Kentaro K. Shimizu

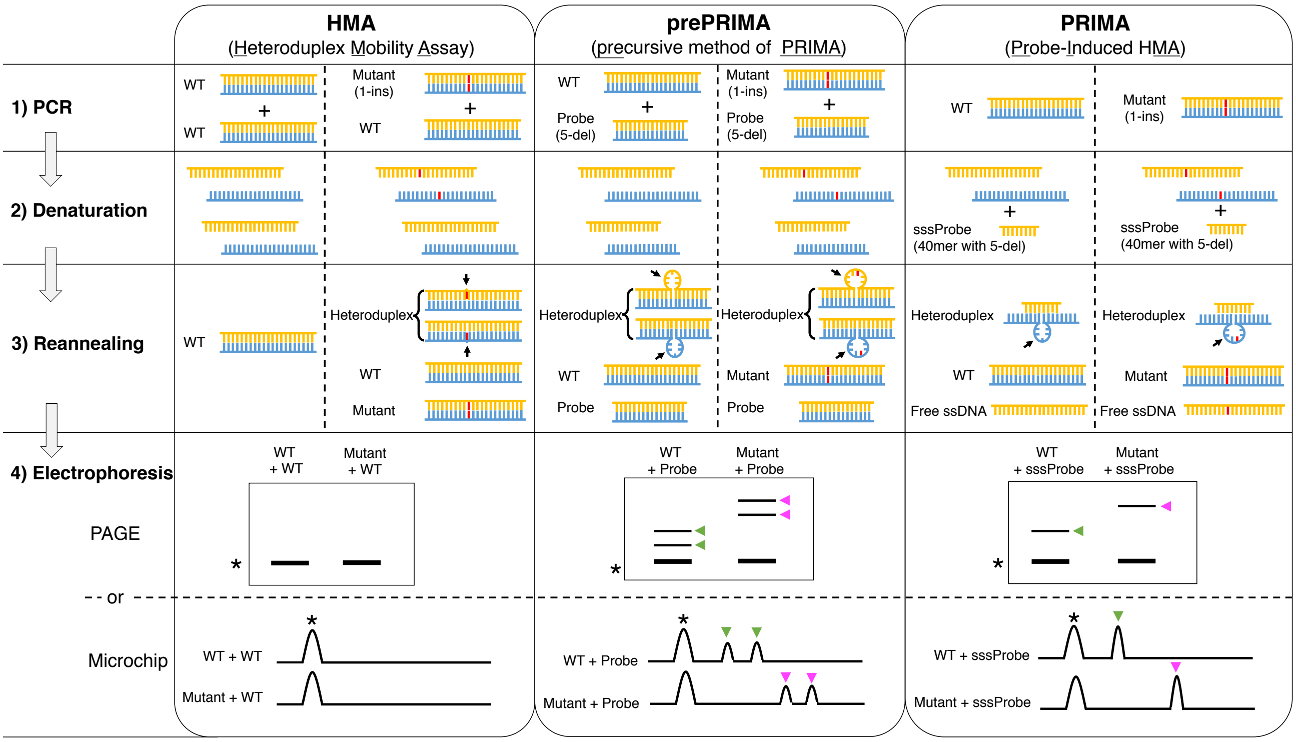

Supplementary Figure S1 | Overview of HMA, prePRIMA, and PRIMA

A schematic representation of analyses aiming to distinguish a sequence with a 1-bp insertion (1-ins) from the wild type (WT) is shown. All three methods consist of four simple steps: (1) PCR, (2) denaturation, (3) reannealing, and (4) detection. When two different sequences exist in step 3, heteroduplex DNA with a “bulge” structure is produced (the “bulge” is indicated by arrows). Heteroduplex DNA migrates slower than the homoduplex and can be detected using PAGE or a microchip system. However, using HMA (left), no heteroduplex signals are detected because the bulge length is too short to produce the detectable heteroduplex structures. By contrast, using prePRIMA (middle) and PRIMA (right) samples produce clear heteroduplexes and they have different mobilities for different genotypes because each heteroduplex DNA has a different bulge length. prePRIMA uses double-stranded DNA from a PCR product with a 5-bp deletion as a probe, while PRIMA uses a short single-stranded DNA with a 5-nt deletion as a probe (sssProbe, e.g., 40-mer oligonucleotides). WT, wild type; 1-ins, 1-bp insertion, and 5-del, 5-bp deletion. Red lines of PCR product represent the different nucleotides between 1-ins and WT. Green and magenta arrowheads indicate heteroduplex signals derived from the WT and mutant, respectively. Asterisks indicate signals from homoduplex DNA and indistinguishable heteroduplex DNA (in HMA), probe (in prePRIMA), or free single-strand DNA (in PRIMA). In prePRIMA and PRIMA, note that the schematic figures of PAGE and microchip represent simplified pictures, although more than a single extra signal possibly due to more complex DNA structure may be observed (see text).

a

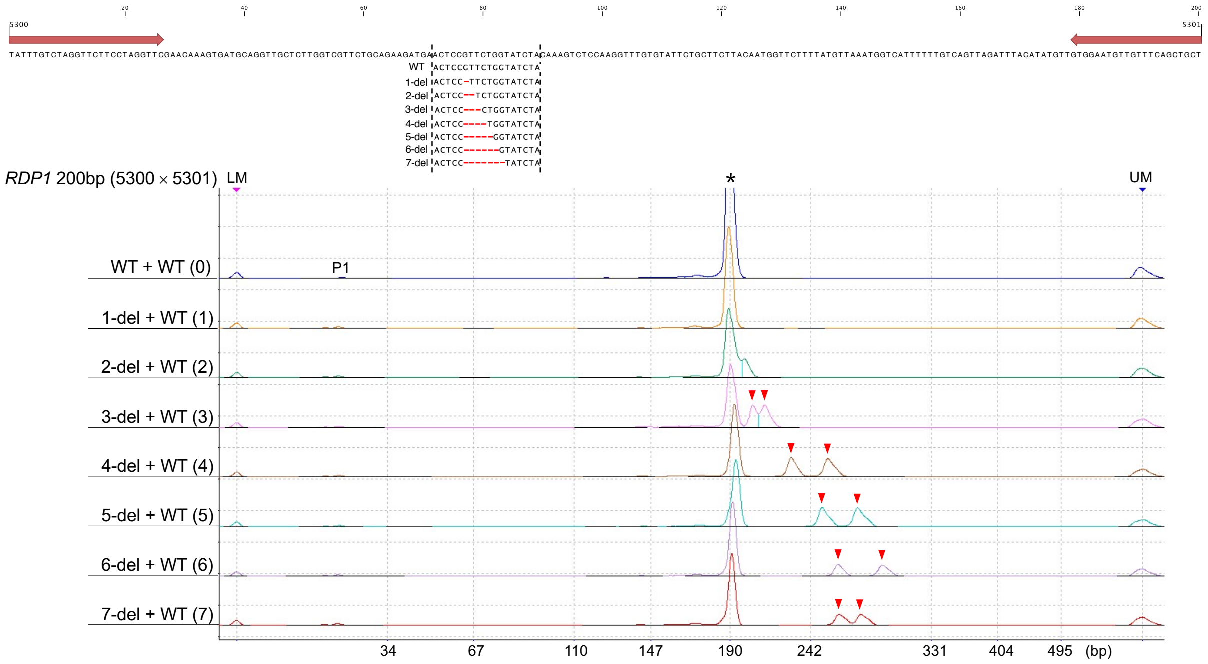

b

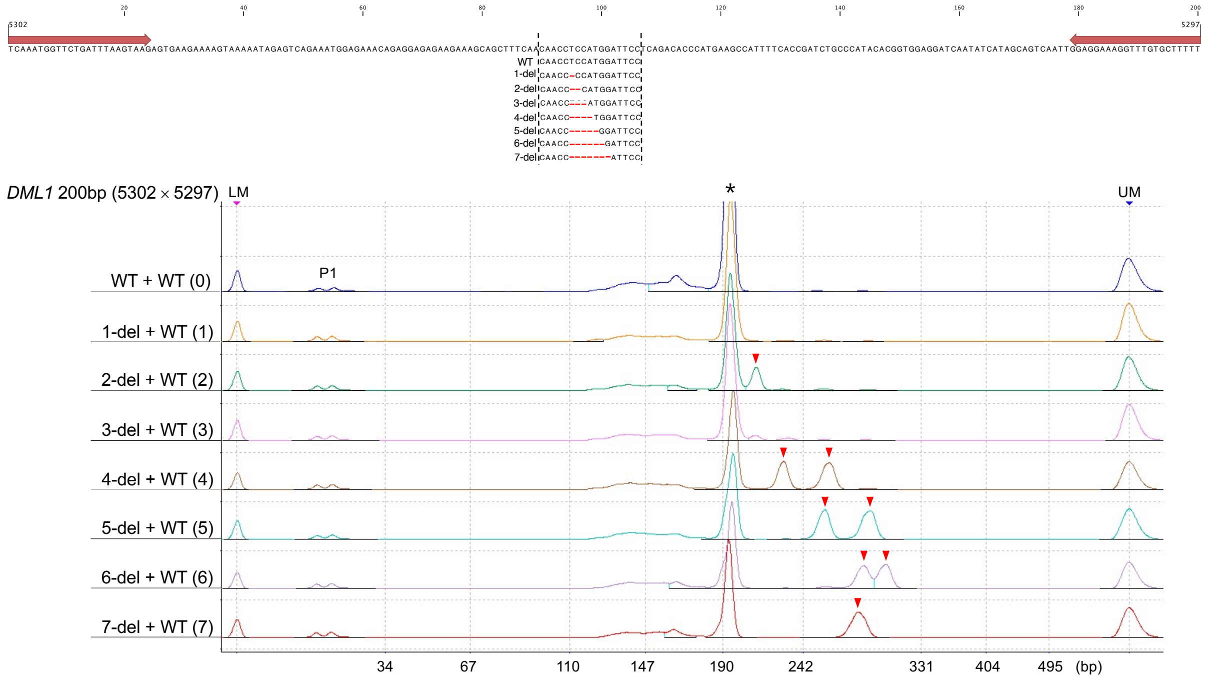

Supplementary Figure S2 | Detection of heteroduplex peaks from WT to 7-del sequence series by HMA.

Partial DNA sequence information and electropherogram by microchip analysis of two genes, (a) *REDUCED POLLEN NUMBER 1*[45] (*RDP1*) and (b) *DEMETER-LIKE 1*[46] (*DML1*) of *Arabidopsis thaliana*. Red arrows indicate primer regions and directions of forward or reverse of the gene of interest. Four digits (e.g., 5300) indicate primer ID. Primer and target sequences are shown in Supplementary Table S1. Primer combinations are shown in the bracket as primer 1 × primer 2 (e.g., 5300 × 5301 in (a)). Electropherograms by microchip analysis using WT and 1-bp to 7-bp

deletion (1-del to 7-del) sequences are shown. In all the electropherograms in this article, X-axes indicate DNA size estimated automatically using markers and the ladder pUC19/*Msp*I (not shown), Y-axes represent signal intensity. LM and UM indicate lower and upper markers, respectively. P1 indicates primer peaks. For the mixture of 1-del and WT target fragments (1-del + WT), no heteroduplex peaks were produced in either gene, *RDP1* (a) and *DML1* (b). Nor did the mixture of 2-del and WT sequences (2-del + WT) in *RDP1* (a) or 3-del and WT sequences (3-del + WT) in *DML1* (b) induce detectable shifts from WT signals. By contrast, shifted heteroduplex peaks were detected from 4-bp to 6-bp (“4-del + WT”, “5-del + WT”, and “6-del + WT”) in each gene because they have sufficient bulge length. Sample information about the mixtures is written on the left side of electropherograms. Brackets next to the sample information indicate bulge length between sample and WT. Red arrowheads indicate heteroduplex peaks. Asterisks indicate homoduplex peaks or indistinguishable heteroduplex peaks. Dashed lines in the chromatograms are automatically embedded by the software.

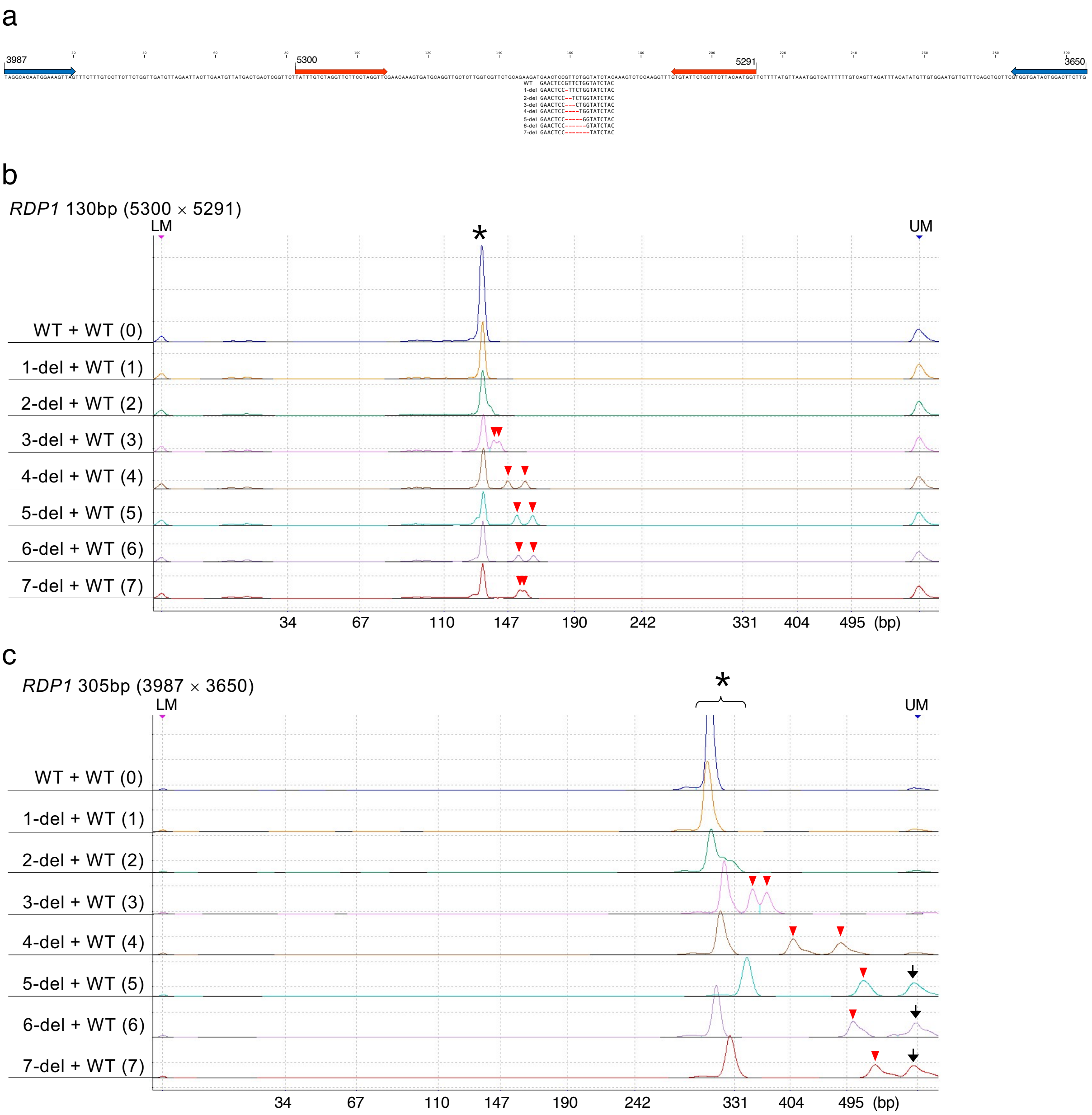

Supplementary Figure S3 | Detection of heteroduplex peaks from WT to 7-del sequence series by HMA using different target fragment sizes.

(a) The partial DNA sequence of *RDP1* and primer binding positions are shown. Red and blue arrows indicate primer binding sites for the target fragment of 130-bp in (b) and 305-bp in (c), respectively. (b) In the case of 130-bp target fragments, the shifts of heteroduplex peaks are less prominent than the shift in the case of 200-bp (Supplementary Fig. S2a). (c) Heteroduplex signals were occasionally misrecognized as upper marker (UM) in 305-bp target fragments (5-del + WT, 6-del + WT, 7-del + WT, black arrows). Primer and target sequences are shown in Supplementary Table S1. Red arrowheads indicate heteroduplex peaks. Asterisks indicate homoduplexes. Brackets next to the sample information indicate bulge length between sample and WT.

a

AT1G25270 (*Arabidopsis thaliana*) 242bp (5373 × 4884)

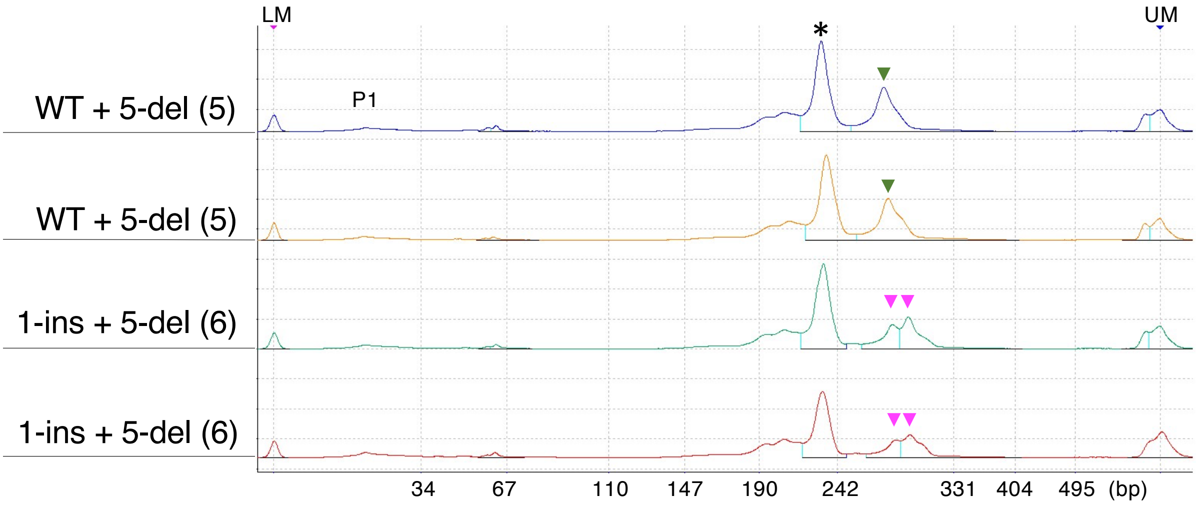

b

AT2G24440 (*Arabidopsis thaliana*) 236bp (3403 × 3389)

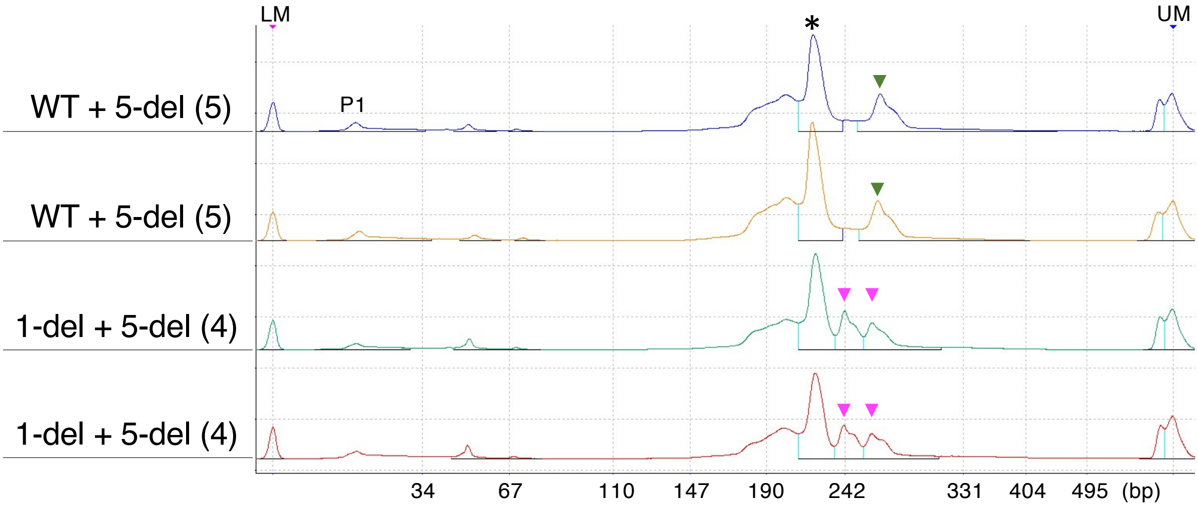

c

AT5G01250 (*Arabidopsis thaliana*) 250bp (5370 × 5372)

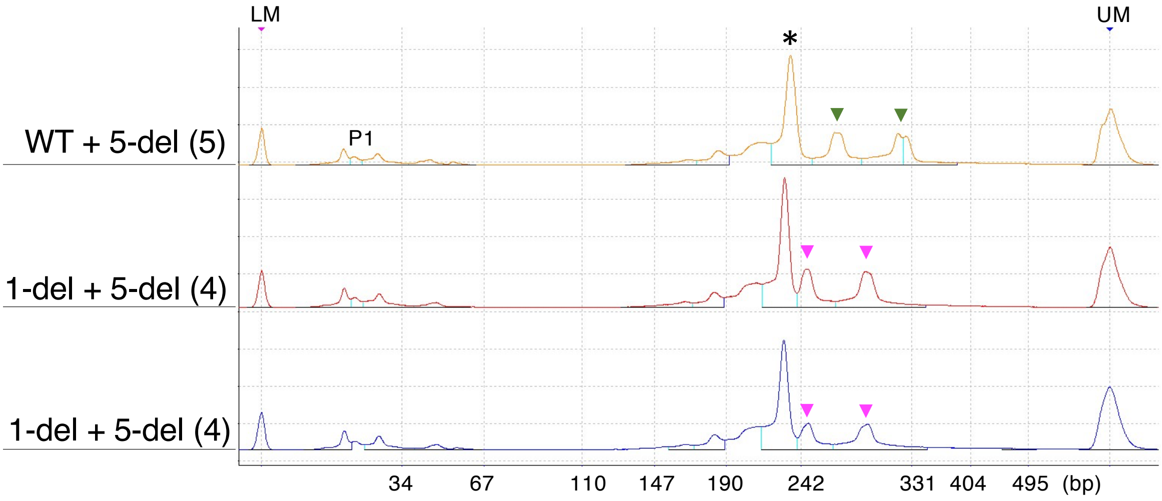

d

Ampicillin resistance (*bla*) gene (Bacterium) 250bp (5363 × 5364)

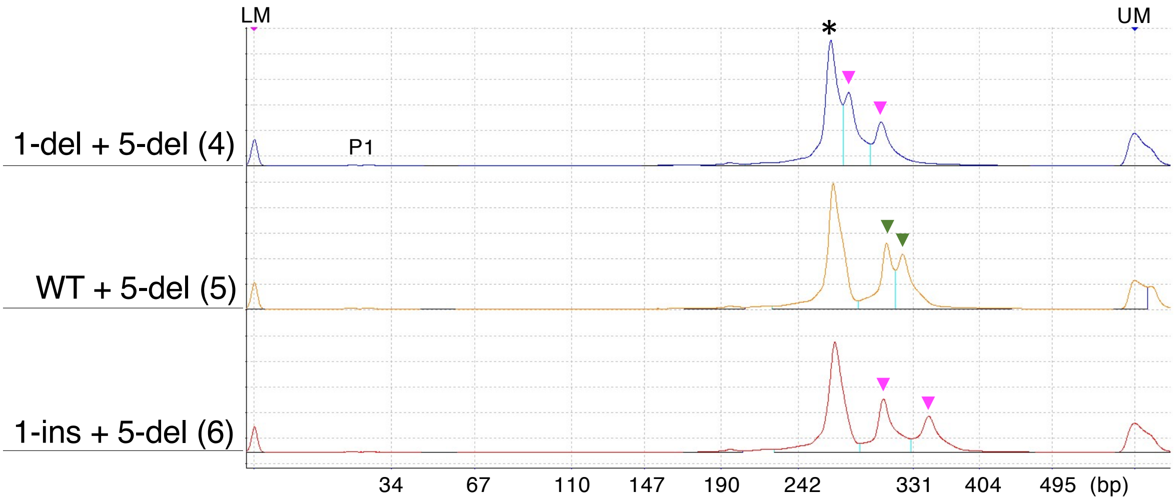

e

Alcohol dehydrogenase 1B (Human) 250bp (5357 × 5358)

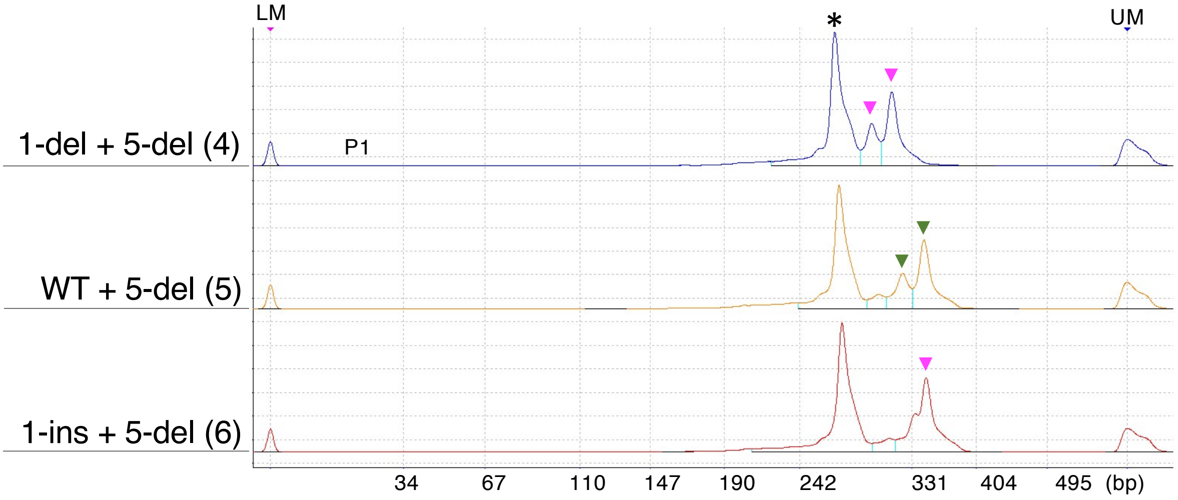

Supplementary Figure S4 | prePRIMA detected different heteroduplex peaks derived from the wild-type and 1-bp indel mutant sequences.

PCR fragments amplified from genes of a plant (a, b, and c), bacterium (d), and human (e) were tested. Green and magenta arrowheads indicate heteroduplex peaks derived from the wild-type and mutant sequences, respectively. Primer, probe, and target sequences are shown in Supplementary Table S1. Brackets next to the sample information indicate bulge length between the target DNA and probe. Asterisks indicate homoduplex peaks or indistinguishable heteroduplex peaks. P1 peak is considered to be leftover primers from the PCR reaction. Unmarked peaks are nonspecific.

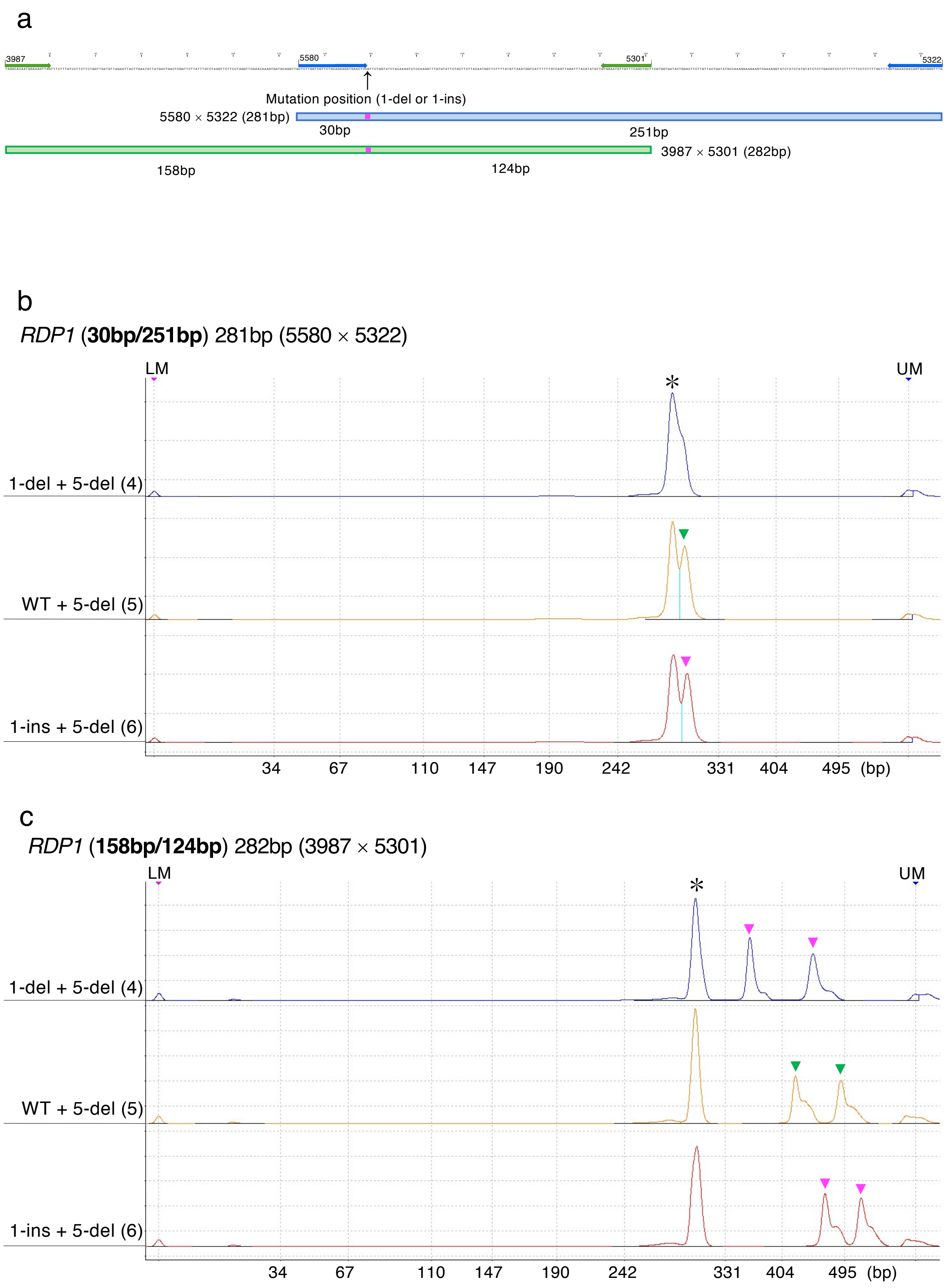

Supplementary Figure S5 | Effect of bulge position on heteroduplex mobility shift patterns in prePRIMA.

(a) Partial *RDP1* sequence. Arrows indicate primer regions and squares indicate the PCR product regions used in b and c, respectively. Blue and green colors correspond to the primers and amplified PCR products by the primers. Primer, probe, and target sequences are shown in Supplementary Table S1. The mutation

position is shown by the black arrow. (b and c) Nucleotide lengths from the edge to the mutation position are shown in the brackets after the gene name (e.g., 30bp/251bp). (b) No heteroduplex peak (1-del + 5-del) or a smaller mobility shift ("WT + 5-del" and "1-ins + 5-del") was detected when the mutation position was close to the end of the PCR fragments. Nucleotide lengths from the edge to the mutation position are shown in the brackets. (c) Clear heteroduplex peaks with mobility shifts are detected when the mutation position is close to the center of the target fragments. Green and magenta arrowheads indicate heteroduplex peaks from the wild-type and mutant sequences, respectively. Brackets next to the sample information indicate the bulge length between the target DNA and the probe. Asterisks indicate a homoduplex peak or indistinguishable heteroduplex peaks.

a

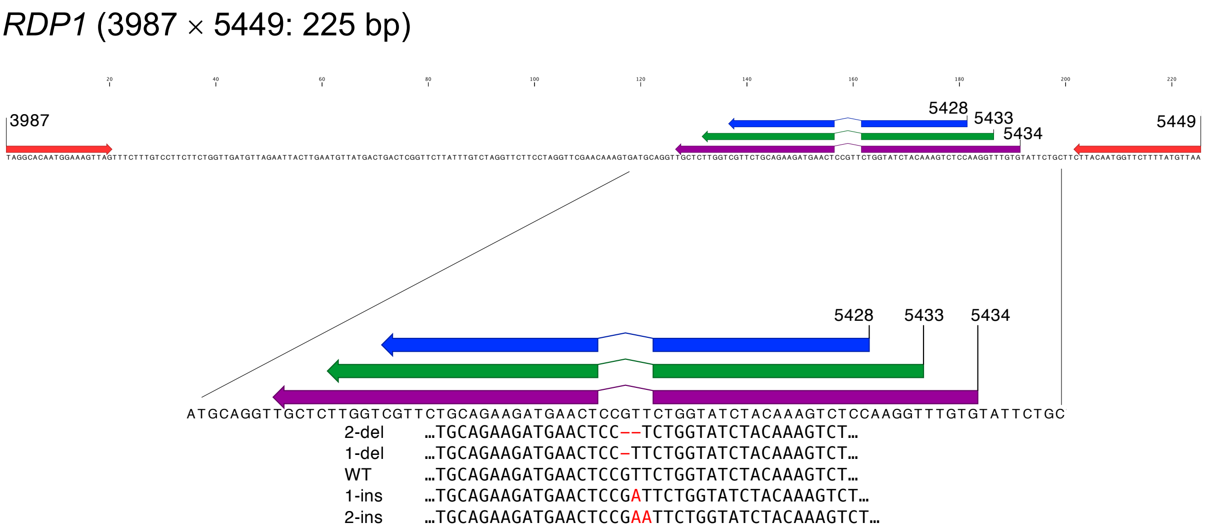

RDP1 (3987 × 5449: 225 bp)
3987
5449
5428
5433
5434
ATGCAGGTTGCTCTTGGTCGTTCTGCAGAAGATGAACTCCGTTCTGGTATCTACAAAGTCTCCAAGGTTTGTGTATTCTGC
2-del …TGCAGAAGATGAACTCC--TCTGGTATCTACAAAGTCT…
1-del …TGCAGAAGATGAACTCC-TTCTGGTATCTACAAAGTCT…
WT …TGCAGAAGATGAACTCCGTTCTGGTATCTACAAAGTCT…
1-ins …TGCAGAAGATGAACTCCGATTCTGGTATCTACAAAGTCT…
2-ins …TGCAGAAGATGAACTCCGAATTCTGGTATCTACAAAGTCT…

b

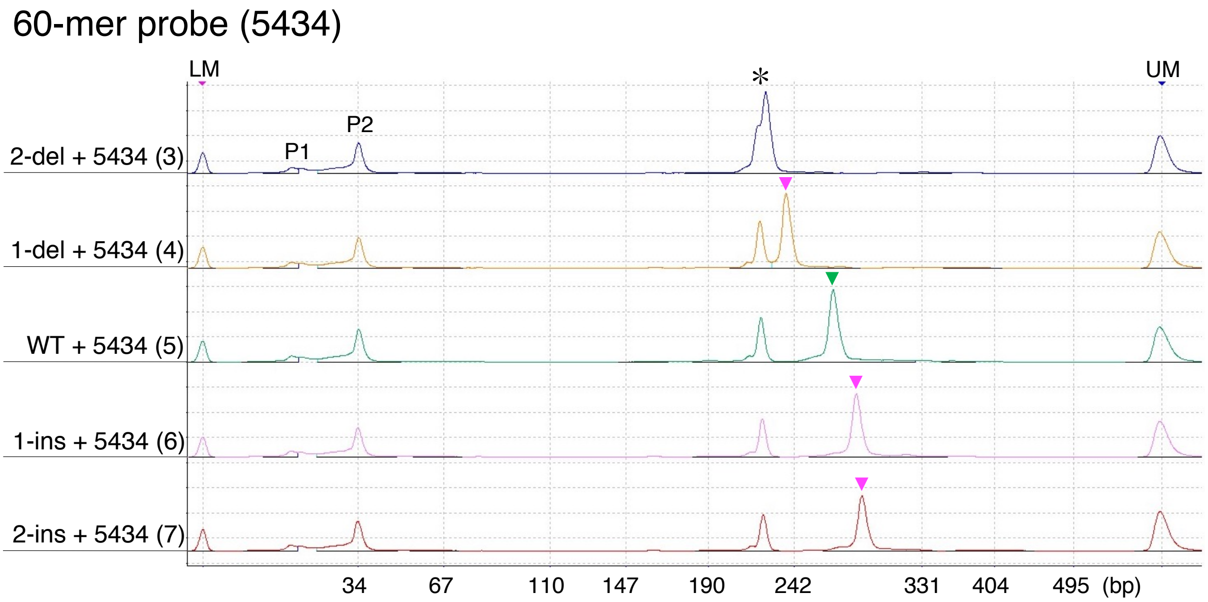

60-mer probe (5434)
LM
UM
*
P1
P2
2-del + 5434 (3)
1-del + 5434 (4)
WT + 5434 (5)
1-ins + 5434 (6)
2-ins + 5434 (7)
34
67
110
147
190
242
331
404
495
(bp)

c

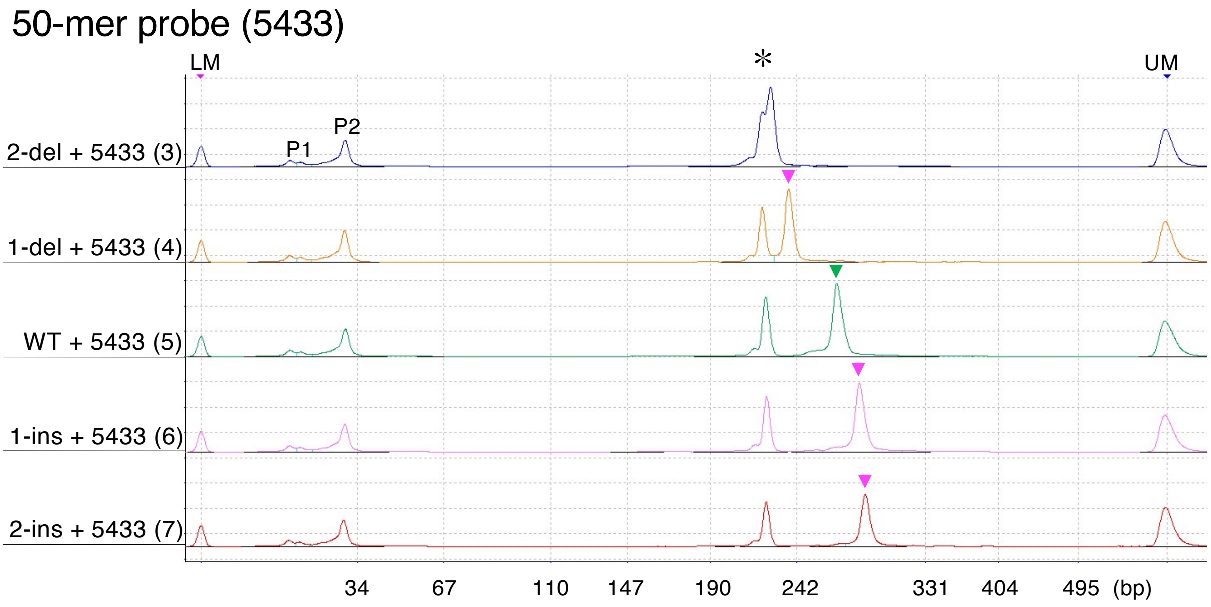

50-mer probe (5433)
LM
UM
*
P1
P2
2-del + 5433 (3)
1-del + 5433 (4)
WT + 5433 (5)
1-ins + 5433 (6)
2-ins + 5433 (7)
34
67
110
147
190
242
331
404
495
(bp)

d

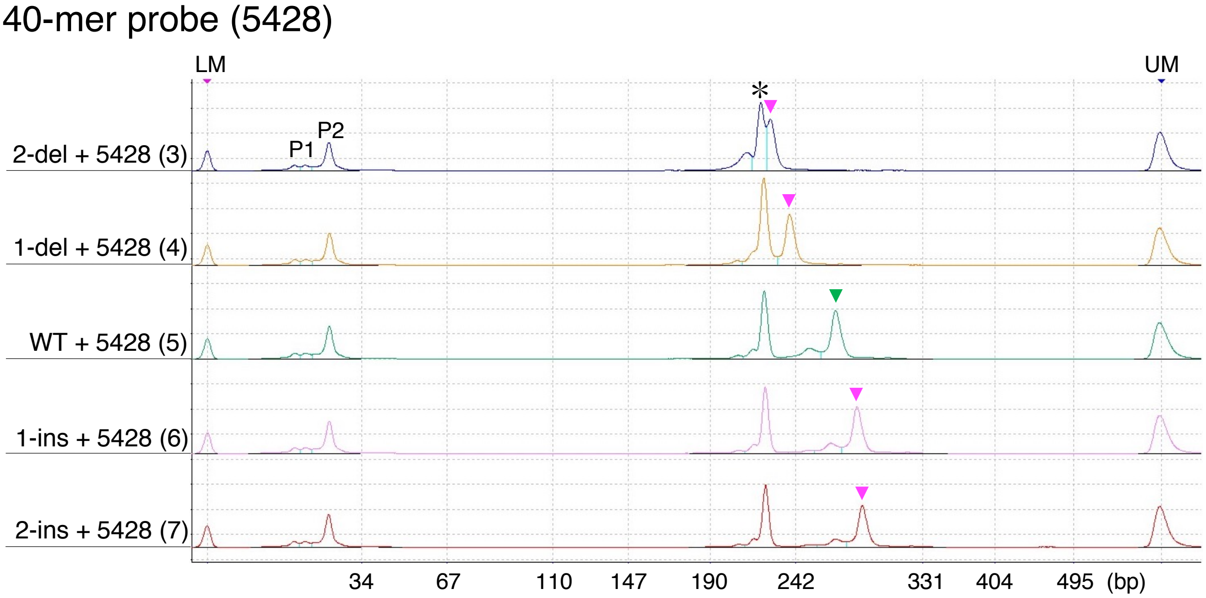

Supplementary Figure S6 | Heteroduplex peak patterns with different lengths of the single-strand probe.

(a) Partial *RDP1* sequence. Red arrows indicate primer binding sites. Purple, green, and blue arrows indicate the probe region with 60-mer (probe 5434), 50-mer (probe 5433), and 40-mer (probe 5428) length, respectively. Primer, probe, and target sequences are shown in Supplementary Table S1. (b–d) Similar heteroduplex peak patterns were produced with different probe lengths. The signal strength of the heteroduplex tended to be higher using a longer probe. Asterisks indicate homoduplex peaks. Green and magenta arrowheads indicate heteroduplex peaks from the wild-type or mutant sequences, respectively. Brackets next to the sample information indicate the bulge length between the target DNA and probe. P1 and P2 peaks are considered to be leftover primers from the PCR reaction and the unbound probe, respectively.

Ampicillin resistance (*bla*) gene (5608 × 5609: 200 bp)

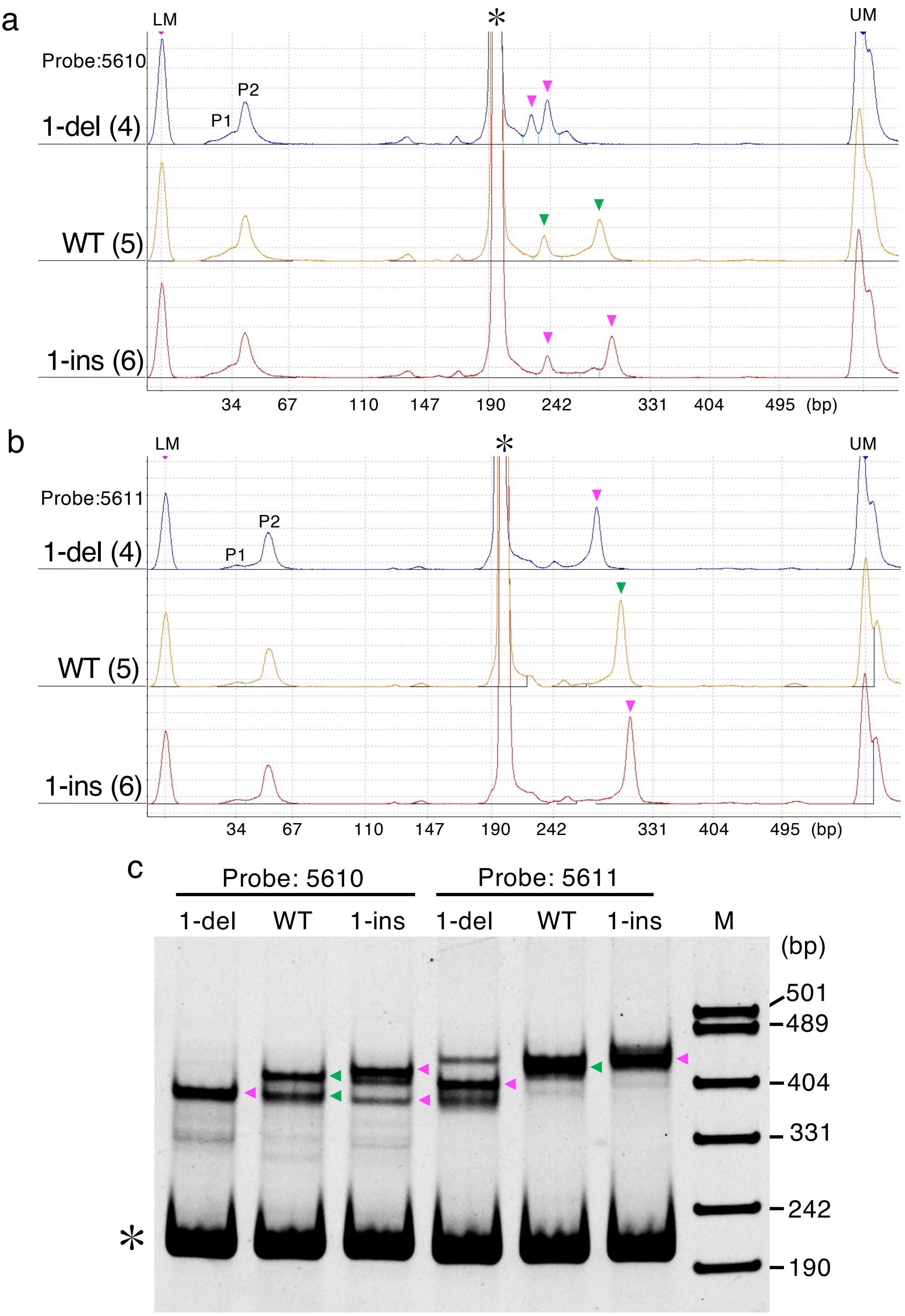

Alcohol dehydrogenase 1B (5555x5556: 200 bp)

d

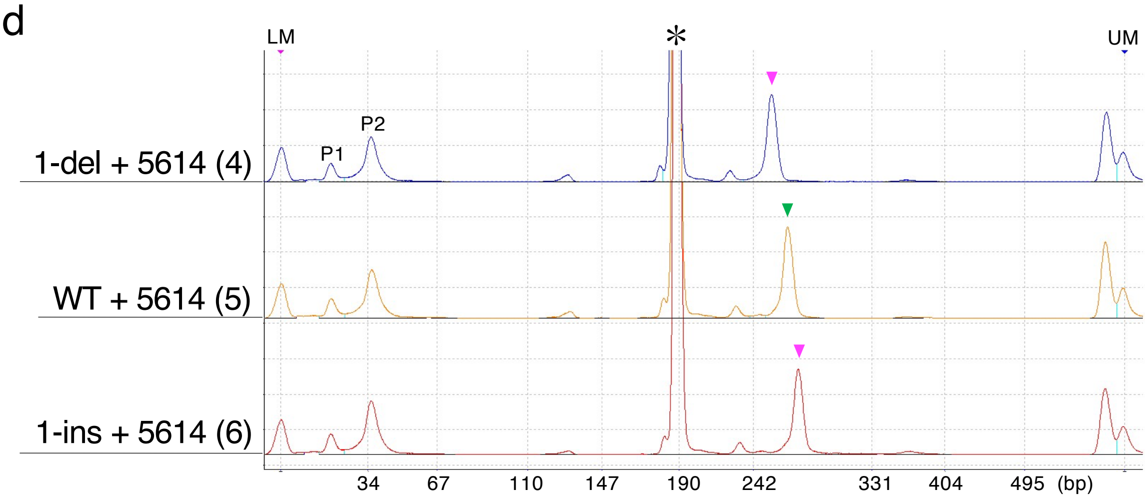

e

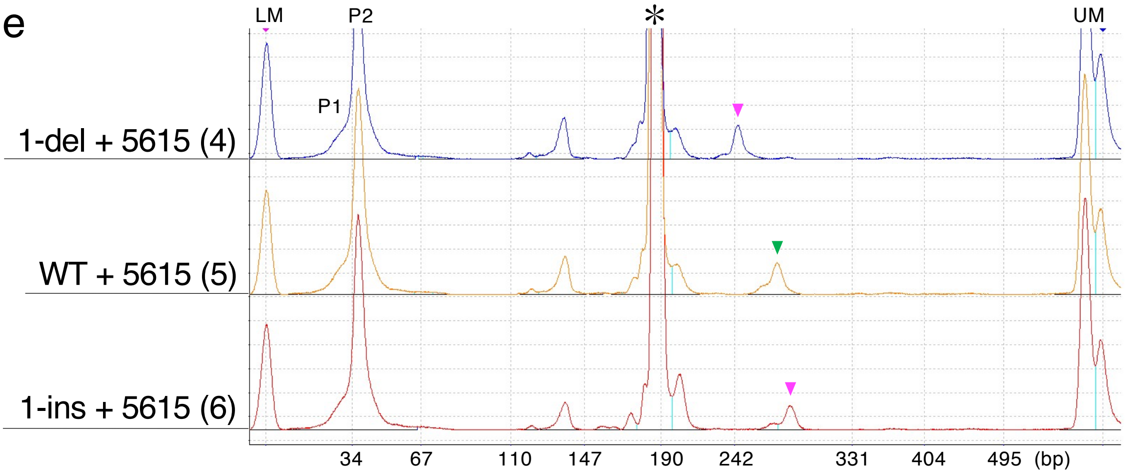

f

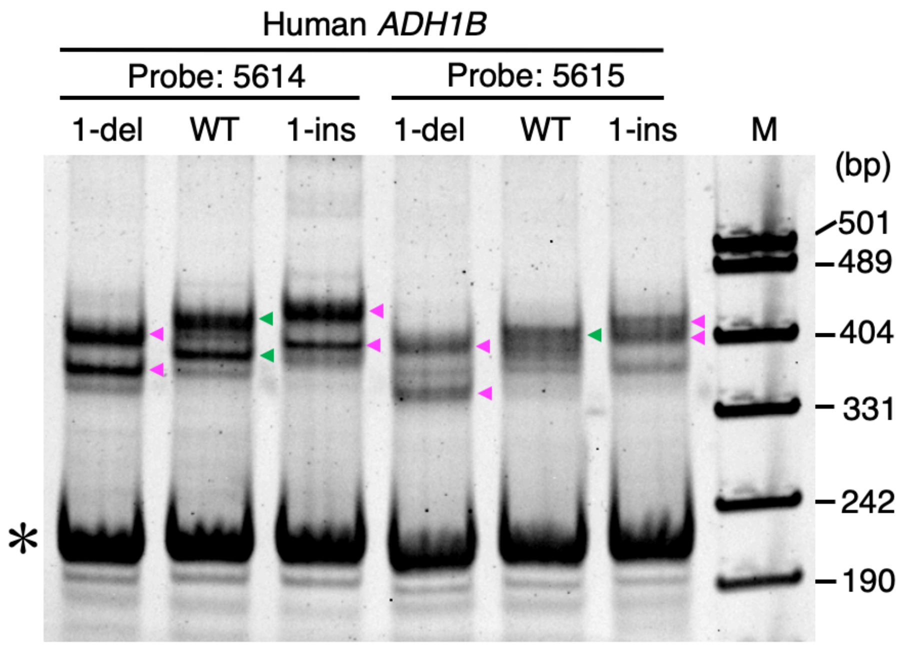

AT1G25270 (5373 × 4884: 242 bp)

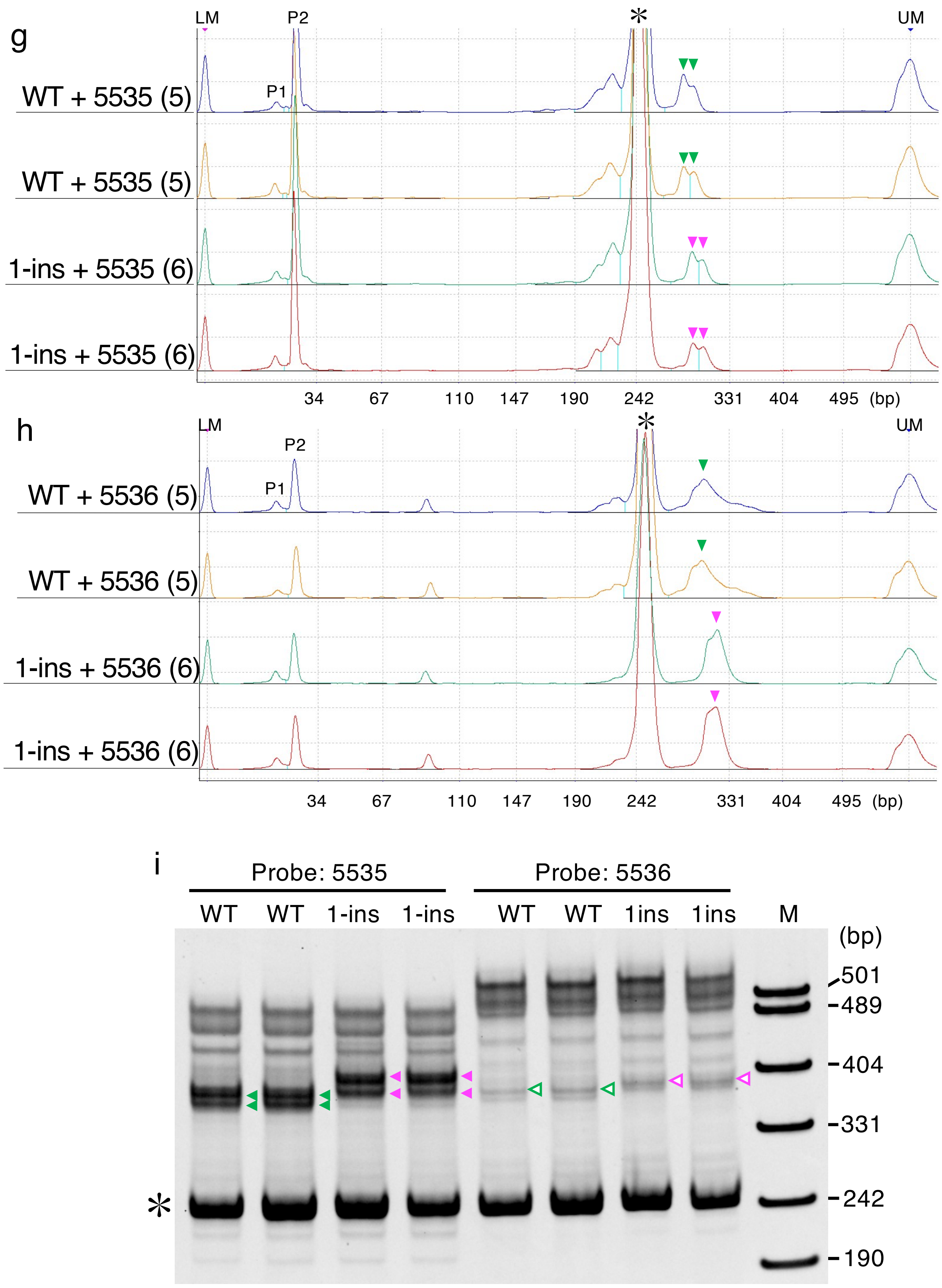

AT2G24440 (5563 × 5564: 250 bp)

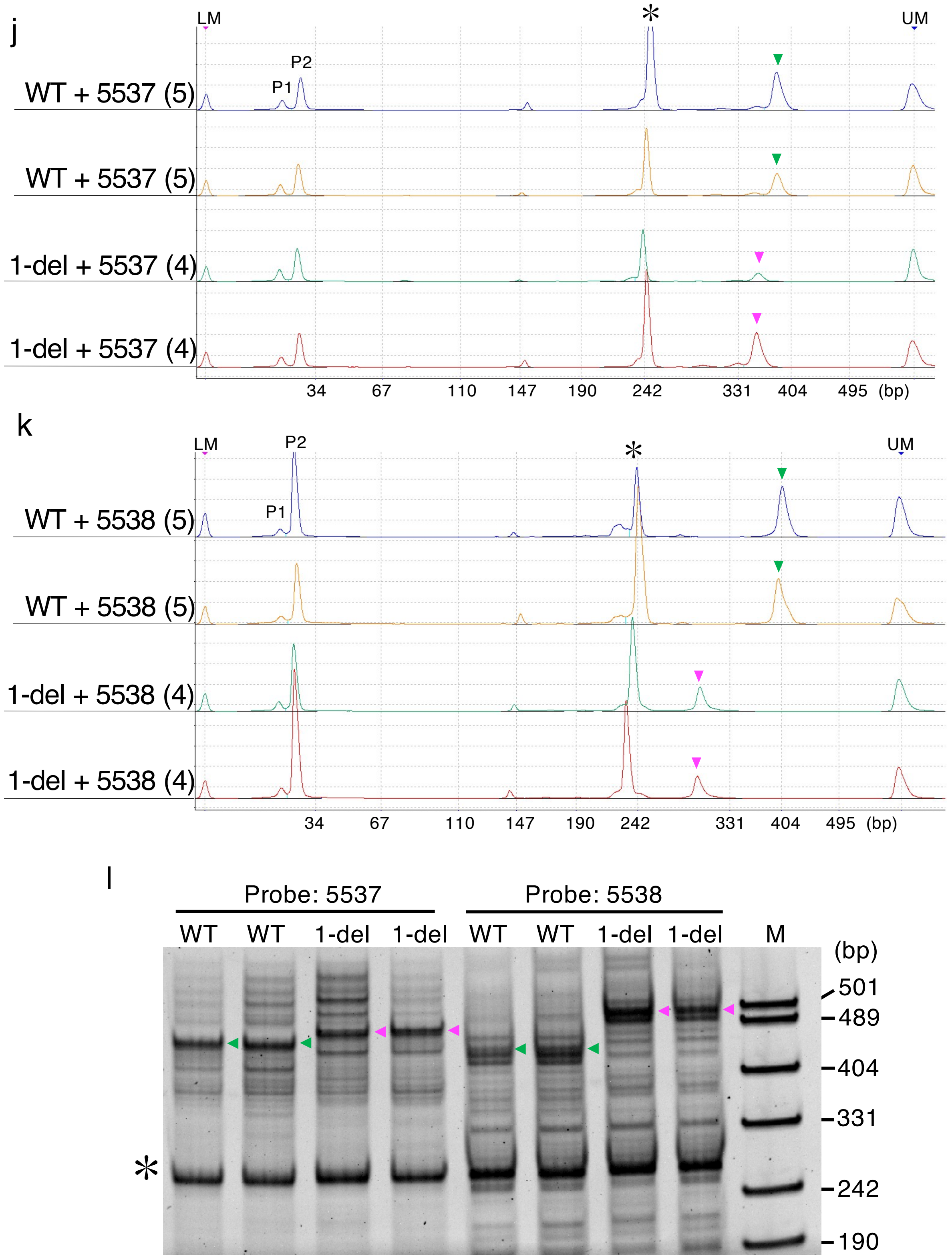

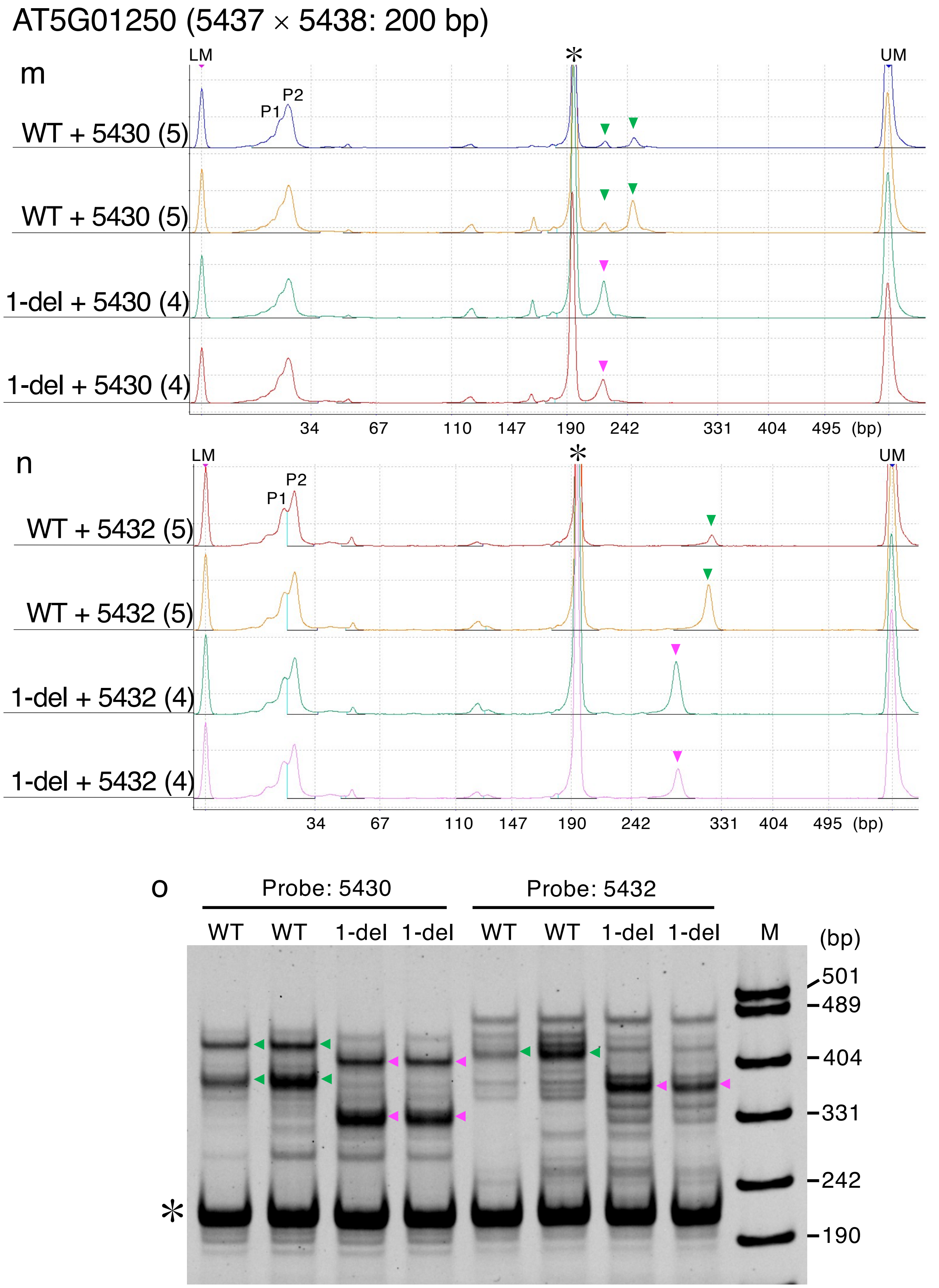
AT5G01250 (5437 × 5438: 200 bp)
m
LM
*
UM
P1
P2
WT + 5430 (5)
WT + 5430 (5)
1-del + 5430 (4)
1-del + 5430 (4)
34
67
110
147
190
242
331
404
495
(bp)
n
LM
*
UM
P1
P2
WT + 5432 (5)
WT + 5432 (5)
1-del + 5432 (4)
1-del + 5432 (4)
34
67
110
147
190
242
331
404
495
(bp)
o
Probe: 5430
Probe: 5432
WT
WT
1-del
1-del
WT
WT
1-del
1-del
M
(bp)
501
489
404
331
242
190
*

Supplementary Figure S7 | PRIMA examples with wild-type and 1-bp indel mutants.

The wild-type and 1-bp indel mutant sequences were analyzed using PRIMA (Ampicillin resistance (*bla*) gene, a-c; *Alcohol dehydrogenase 1B,* d-f; AT1G25270, g-i; AT2G24440, j-l; and AT5G01250, m-o). Three mutant sequences from *Arabidopsis thaliana* were produced by CRISPR/Cas9 (g-o). Heteroduplex peaks distinguishable between the wild-type and 1-bp indel mutant sequences were detected in all three sequences although weak heteroduplex bands were detected from one example (i, right). Primer, probe, and target sequences are shown in Supplementary Table S1. Asterisks indicate homoduplex peaks. Green and magenta arrowheads indicate heteroduplex peaks created by the wild-type and mutant sequences, respectively. Brackets next to the sample information indicate the bulge length between the target DNA and the probe. P1 and P2 peaks are considered to be leftover primers from the PCR reaction and the unbound probe, respectively. Nonspecific signals are not labeled.

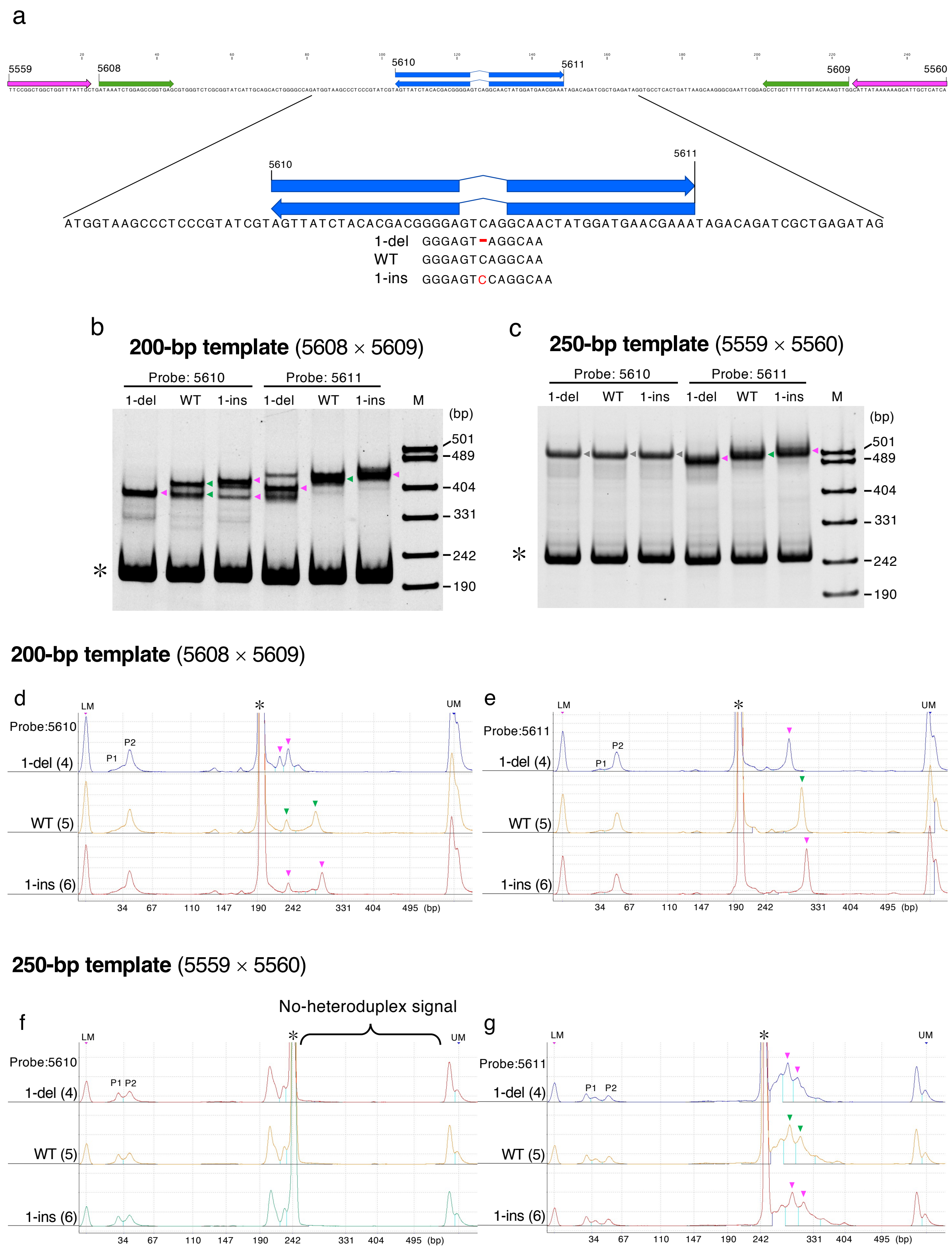

Supplementary Figure S8 | Different target fragment sizes changed heteroduplex signals in the ampicillin resistance gene.

(a) Partial sequence of the ampicillin resistance gene. Green arrows indicate the primer regions used to amplify the 200-bp fragment. Magenta arrows indicate the primer region used to amplify the 250-bp fragment. Blue arrows indicate probe regions with a 5-nt deletion. Mutation sequences are shown in red text. Clear

heteroduplex signals were produced by 200-bp target DNA fragments (b, d, e) from both complementary probes (5610 and 5611). By contrast, in 250-bp target DNA fragments, indistinguishable bands (shown by gray arrowheads in c, left) or no heteroduplex peaks (f) were detected using 5610 probes, although there were clear heteroduplex signals from 5611 probes (c right and g). Results from the 200-bp template DNA were the same as Supplementary Fig. S7a–c and shown here for comparison. Primer, probe, and target sequences are shown in Supplementary Table S1. Brackets next to the sample information indicate bulge length between target DNA and probe. P1 and P2 peaks are considered to be leftover primers from the PCR reaction and the unbound probe, respectively. Nonspecific signals are not labeled.

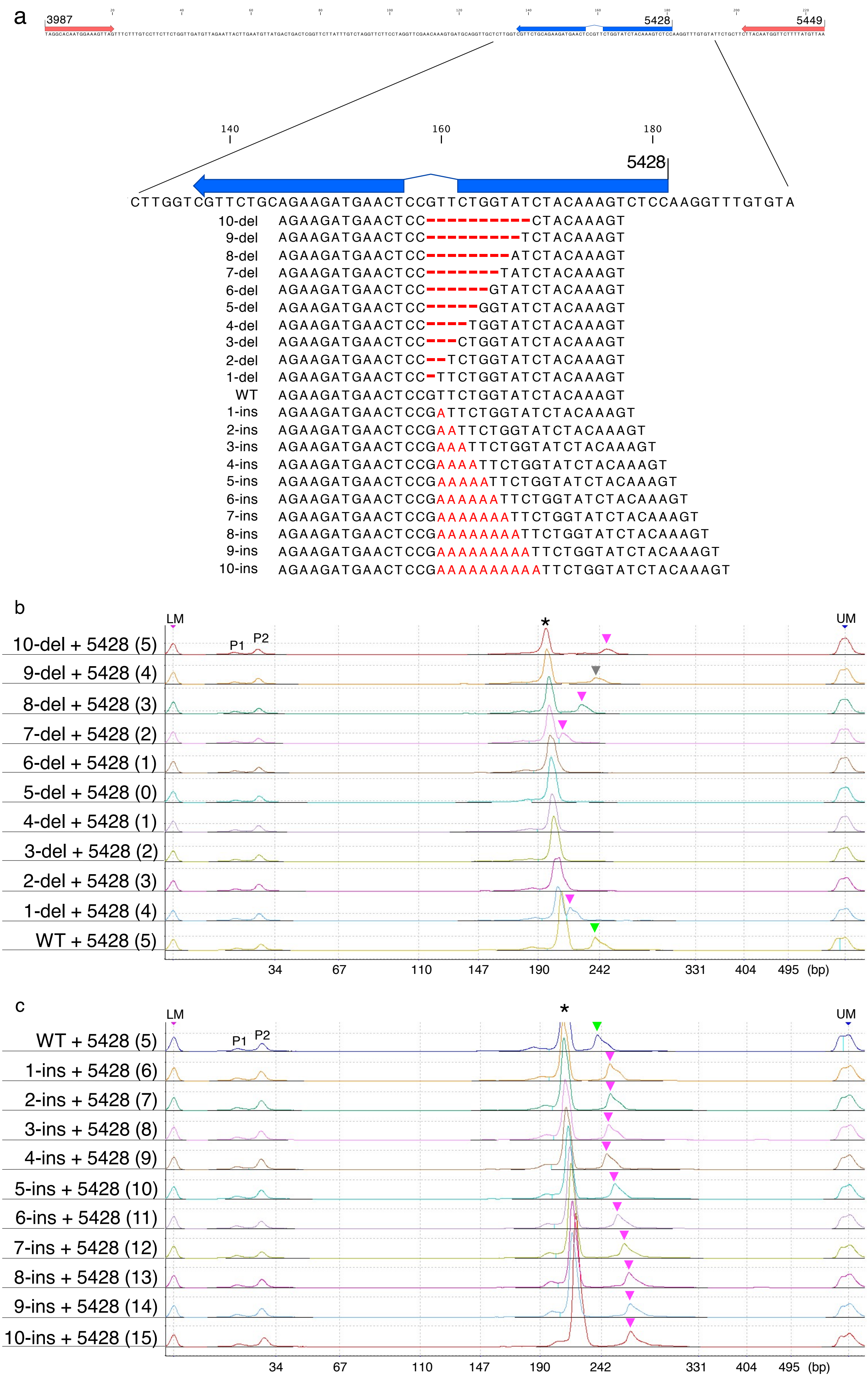
a
3987
5428
5449
140
160
180
5428
CTTGGTCGTTCTGCAGAAGATGAACTCCGTTCTGGTATCTACAAAGTCTCCAAGGTTTGTGTA
10-del AGAAGATGAACTCC----------CTACAAAGT
9-del AGAAGATGAACTCC---------TCTACAAAGT
8-del AGAAGATGAACTCC--------ATCTACAAAGT
7-del AGAAGATGAACTCC-------TATCTACAAAGT
6-del AGAAGATGAACTCC------GTATCTACAAAGT
5-del AGAAGATGAACTCC-----GGTATCTACAAAGT
4-del AGAAGATGAACTCC----TGGTATCTACAAAGT
3-del AGAAGATGAACTCC---CTGGTATCTACAAAGT
2-del AGAAGATGAACTCC--TCTGGTATCTACAAAGT
1-del AGAAGATGAACTCC-TTCTGGTATCTACAAAGT
WT AGAAGATGAACTCCGTTCTGGTATCTACAAAGT
1-ins AGAAGATGAACTCCGATTCTGGTATCTACAAAGT
2-ins AGAAGATGAACTCCGAATTCTGGTATCTACAAAGT
3-ins AGAAGATGAACTCCGAAATTCTGGTATCTACAAAGT
4-ins AGAAGATGAACTCCGAAAATTCTGGTATCTACAAAGT
5-ins AGAAGATGAACTCCGAAAAATTCTGGTATCTACAAAGT
6-ins AGAAGATGAACTCCGAAAAAATTCTGGTATCTACAAAGT
7-ins AGAAGATGAACTCCGAAAAAAATTCTGGTATCTACAAAGT
8-ins AGAAGATGAACTCCGAAAAAAAATTCTGGTATCTACAAAGT
9-ins AGAAGATGAACTCCGAAAAAAAAATTCTGGTATCTACAAAGT
10-ins AGAAGATGAACTCCGAAAAAAAAAATTCTGGTATCTACAAAGT
b
LM
UM
P1
P2
*
10-del + 5428 (5)
9-del + 5428 (4)
8-del + 5428 (3)
7-del + 5428 (2)
6-del + 5428 (1)
5-del + 5428 (0)
4-del + 5428 (1)
3-del + 5428 (2)
2-del + 5428 (3)
1-del + 5428 (4)
WT + 5428 (5)
34
67
110
147
190
242
331
404
495
(bp)
c
LM
UM
P1
P2
*
WT + 5428 (5)
1-ins + 5428 (6)
2-ins + 5428 (7)
3-ins + 5428 (8)
4-ins + 5428 (9)
5-ins + 5428 (10)
6-ins + 5428 (11)
7-ins + 5428 (12)
8-ins + 5428 (13)
9-ins + 5428 (14)
10-ins + 5428 (15)
34
67
110
147
190
242
331
404
495
(bp)

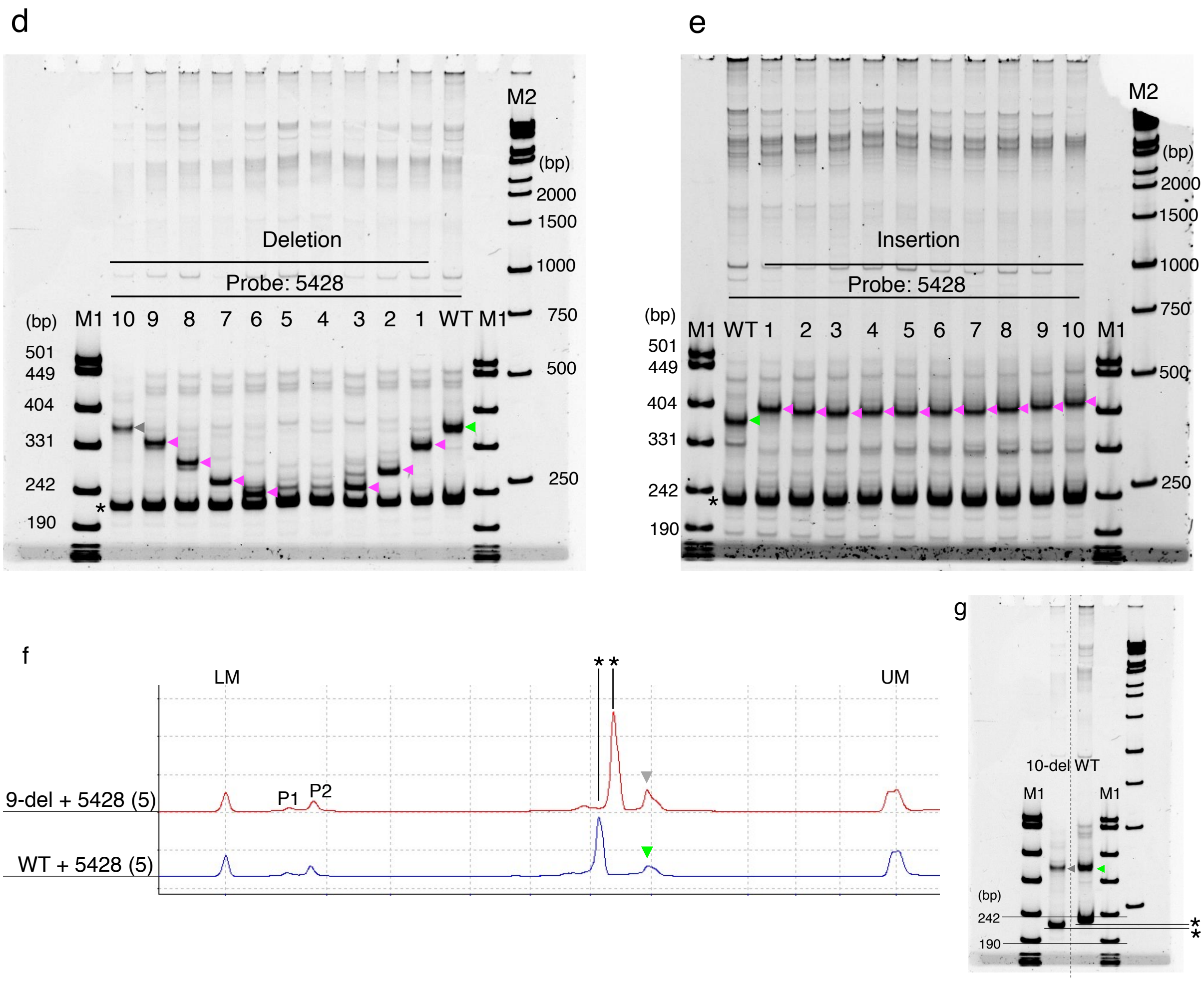

Supplementary Figure S9 | Detection of heteroduplex peaks with sequences with variable lengths of insertions and deletions using PRIMA.

(a) Partial *RDP1* sequence. Red arrows indicate primer regions and the blue arrow indicates the probe region. Arrows also indicate the direction against the gene of interest. Signals were detected using a microchip electrophoresis system (b and c) or polyacrylamide gel electrophoresis (d and e). Heteroduplex peaks were detected from 10-bp deletion to wild-type sequences (b and d) or from the wild-type to 10-bp insertion sequences (c and e). Asterisks indicate a homoduplex signal. Green arrowheads indicate heteroduplex peaks of the wild-type sequence. Magenta arrowheads indicate distinguishable heteroduplex signals of the mutant with mobility shifts from the wild-type sequence. Gray arrowheads indicate heteroduplex signals indistinguishable from the wild-type sequence (9-del in b, 10-del in d). (f and g) Direct comparison between the signals of WT and mutants, which produced indistinguishable heteroduplex signals (WT and 9-del in MultiNA (f); WT and 10-del in PAGE (g)). Their homoduplex signals were distinguishable (asterisks). Note that (f) is an extraction from the same data shown in (a) and (g) is a trimmed gel image of (d). Trimmed line is shown by the break line. Bulge lengths between target DNA and probe are shown in the brackets (b and c). Primer, probe, and target sequences are shown in Supplementary Table S1. WT, wild type; M1 and M2 indicate DNA markers (pUC19/*Msp*I ladder and 1-kbp ladder, respectively). P1 and P2 peaks are considered to be leftover primers from the PCR reaction and the unbound probe, respectively.

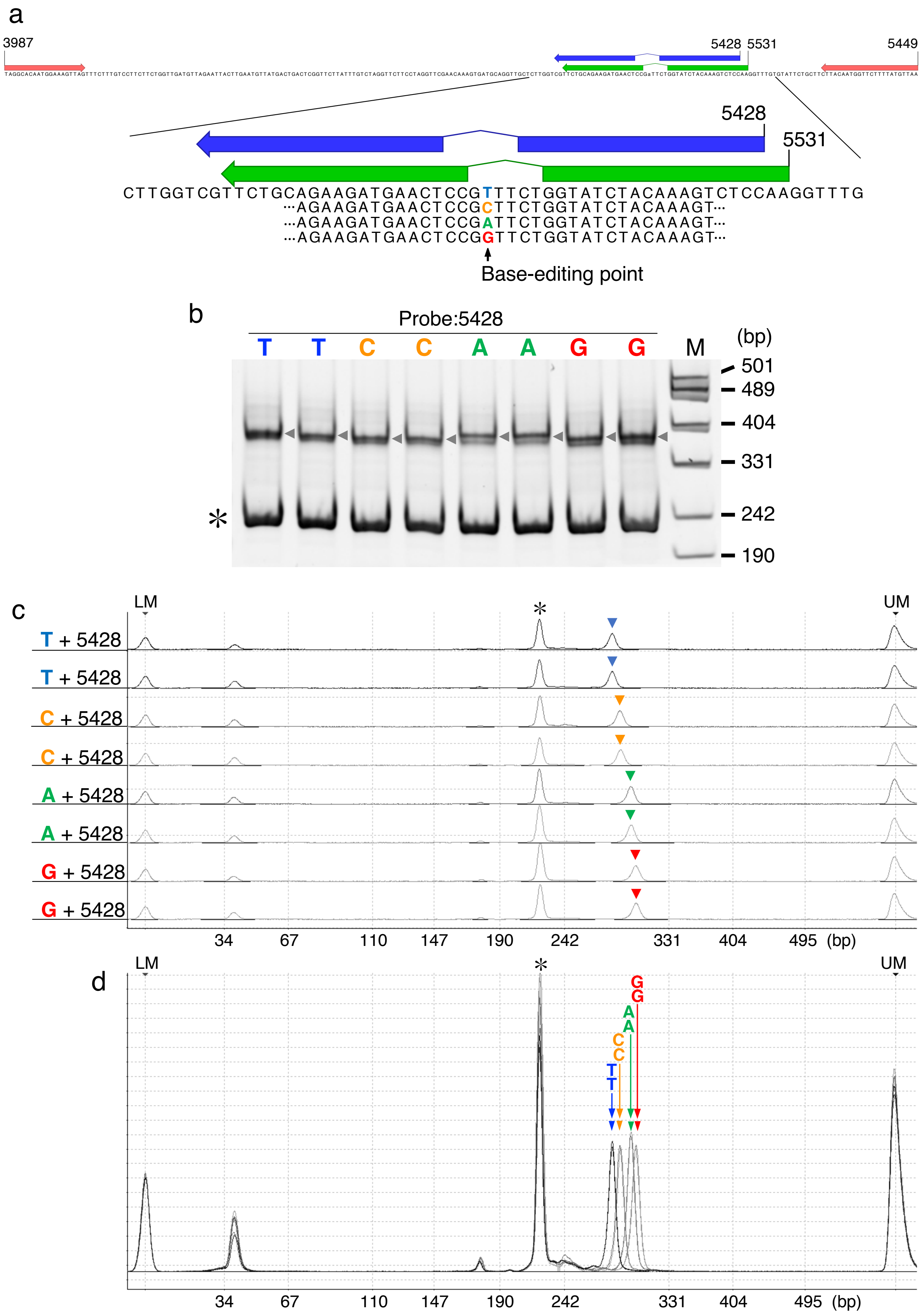
a
3987
5428 5531
5449
5428
5531
CTTGGTCGTTCTGCAGAAGATGAACTCCGTTTCTGGTATCTACAAAGTCTCCAAGGTTTG
···AGAAGATGAACTCCGCTTCTGGTATCTACAAAGT···
···AGAAGATGAACTCCGATTCTGGTATCTACAAAGT···
···AGAAGATGAACTCCGGTTCTGGTATCTACAAAGT···
Base-editing point
b
Probe:5428
T T C C A A G G M
(bp)
501
489
404
331
242
190
*
c
LM
*
UM
T + 5428
T + 5428
C + 5428
C + 5428
A + 5428
A + 5428
G + 5428
G + 5428
34 67 110 147 190 242 331 404 495 (bp)
d
LM
*
UM
G G A A C C T T
34 67 110 147 190 242 331 404 495 (bp)

e

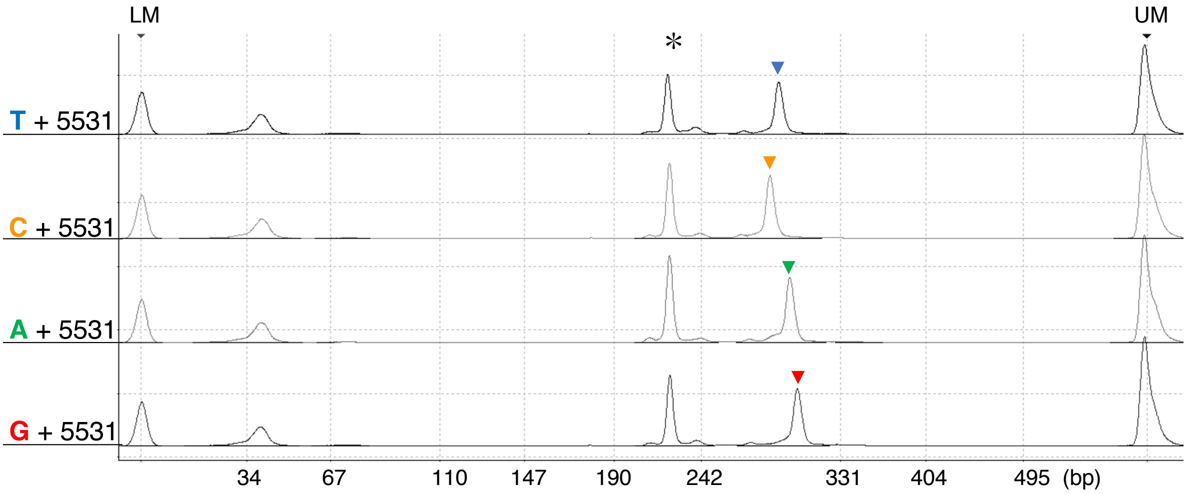

f

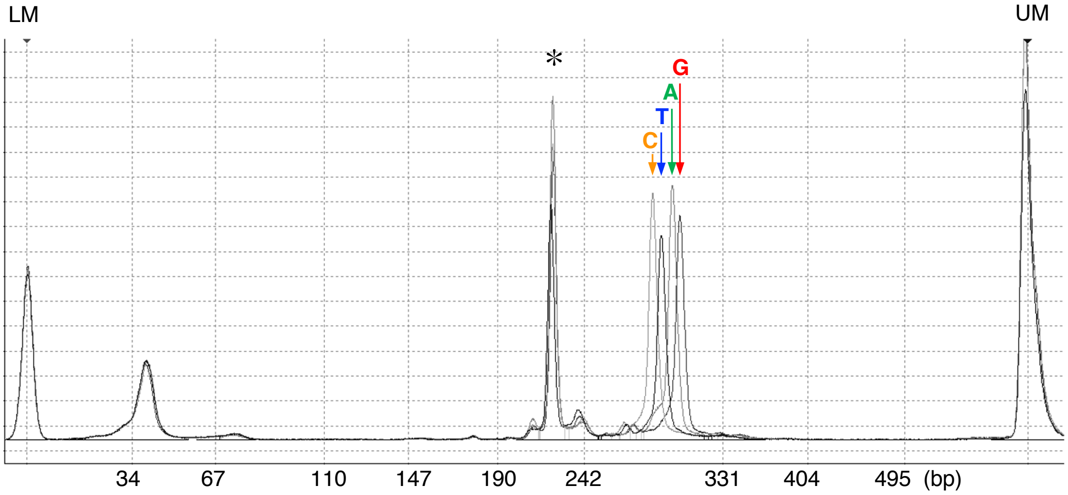

Supplementary Figure S10 | SNP Detection by heteroduplex peak patterns of PRIMA.

Four different SNPs (T, C, A, and G) were prepared in *RDP1* (a). Although unclear results were displayed by PAGE (b), four different heteroduplex peaks were detected by the microchip detected by using 5428 (c and d) and 5531 probes (e and f). (d, f) Chromatographs with four base-editing samples from c and e are overlayed to clearly show the different heteroduplex peak positions. Note that heteroduplex peaks of d contain two technically replicated samples for each nucleotide. Heteroduplex peaks from each base are shown by arrowheads (b, c, e) and arrows (d, f). Asterisks indicate homoduplex peaks. Primer, probe, and target sequences are shown in Supplementary Table S1.

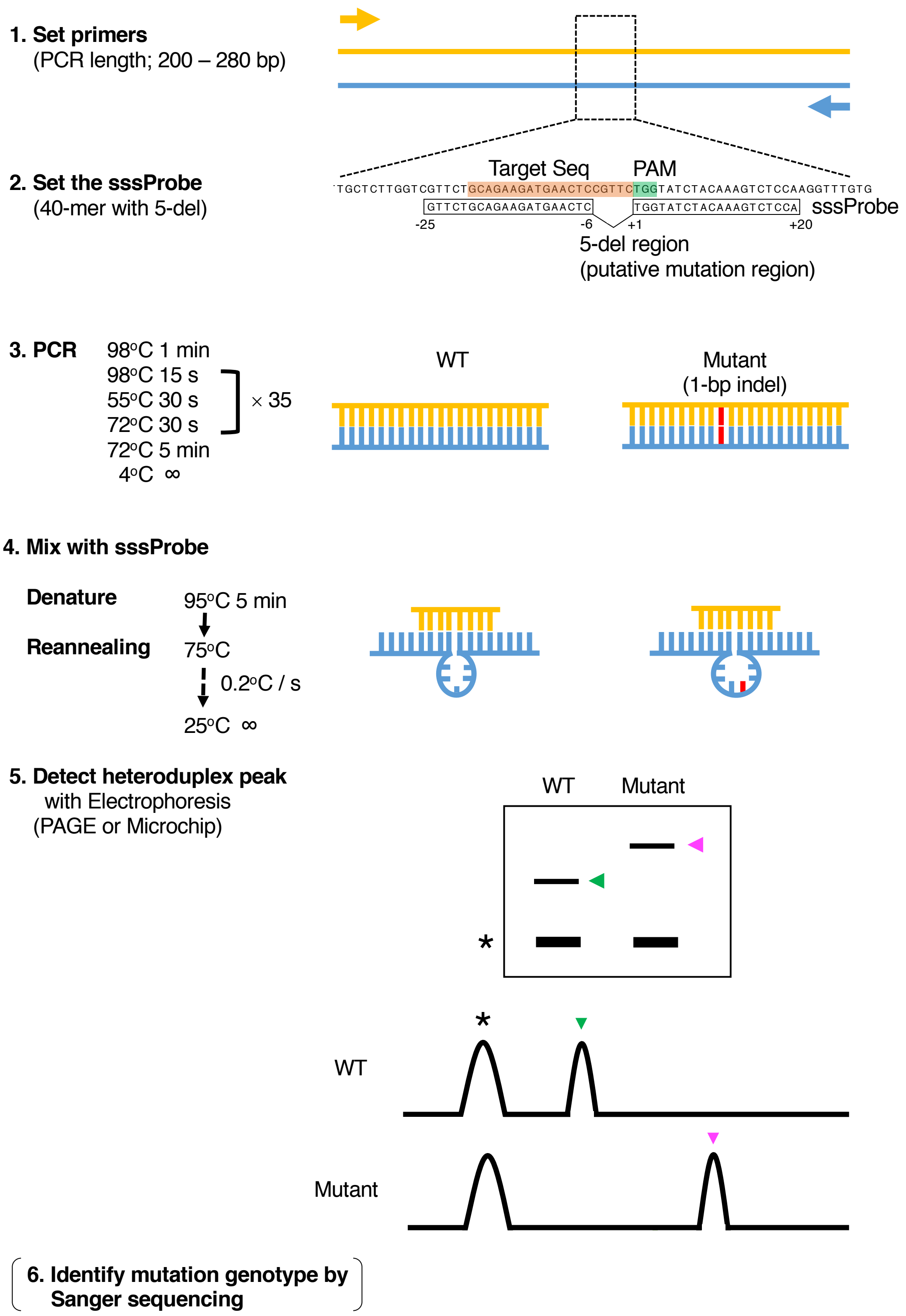

Supplementary Figure S11 | Protocol for genotyping of a CRISPR mutant with PRIMA. Target seq, 20-nt sgRNA binding region of CRISPR/Cas9 system. PAM, the protospacer adjacent motif of the CRISPR system. sssProbe, short single-strand probe. WT, wild type. Asterisk, homoduplex signal. Green and magenta arrowheads, heteroduplex signals from the wild-type and mutant sequences. Details are described in the Methods section.
